# Supplementary material for: GPC: An expressive and tractable deep generative model for genetic variation data
Source: PLoS Genet. 2026 Oct 6;22(10):e1012321. doi: 10.1371/journal.pgen.1012321 (PMC13652338; doi:10.1371/journal.pgen.1012321)
Supplement: Supplementary file 1 [file pgen.1012321.s001.pdf]

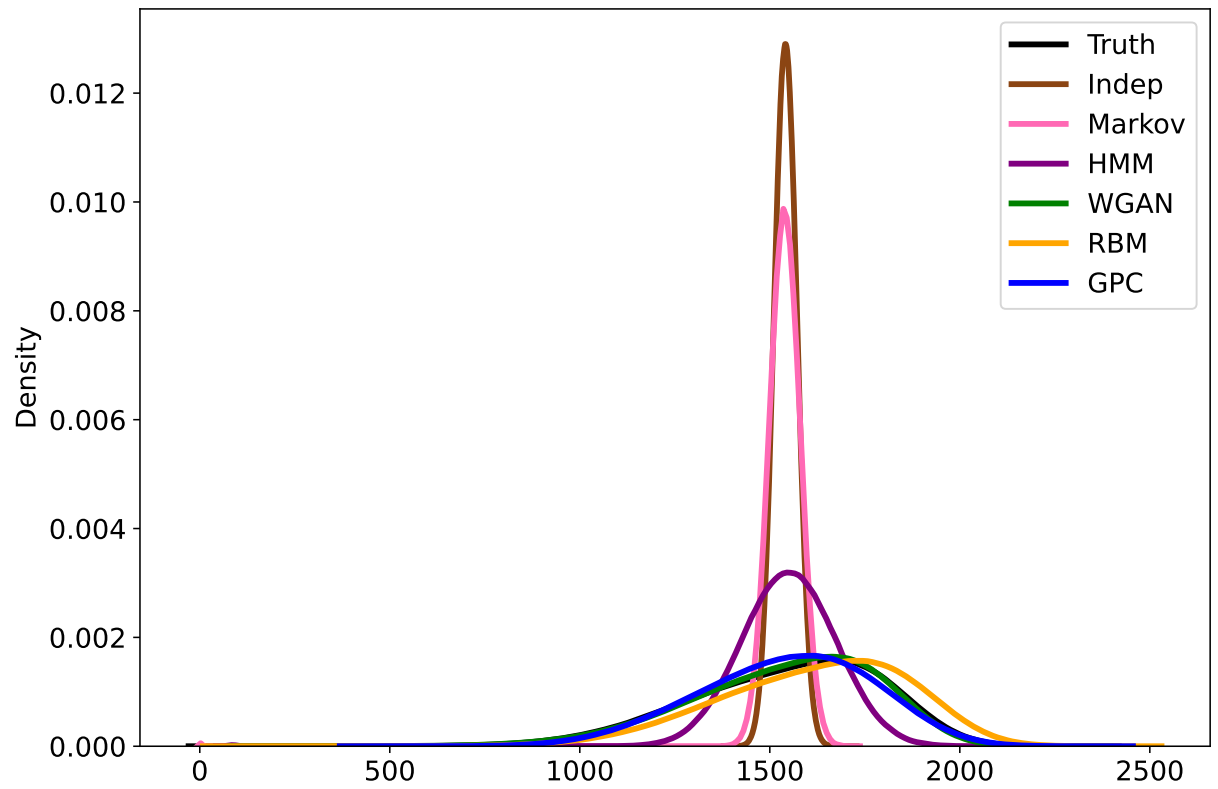

(a) Within each dataset (10K)

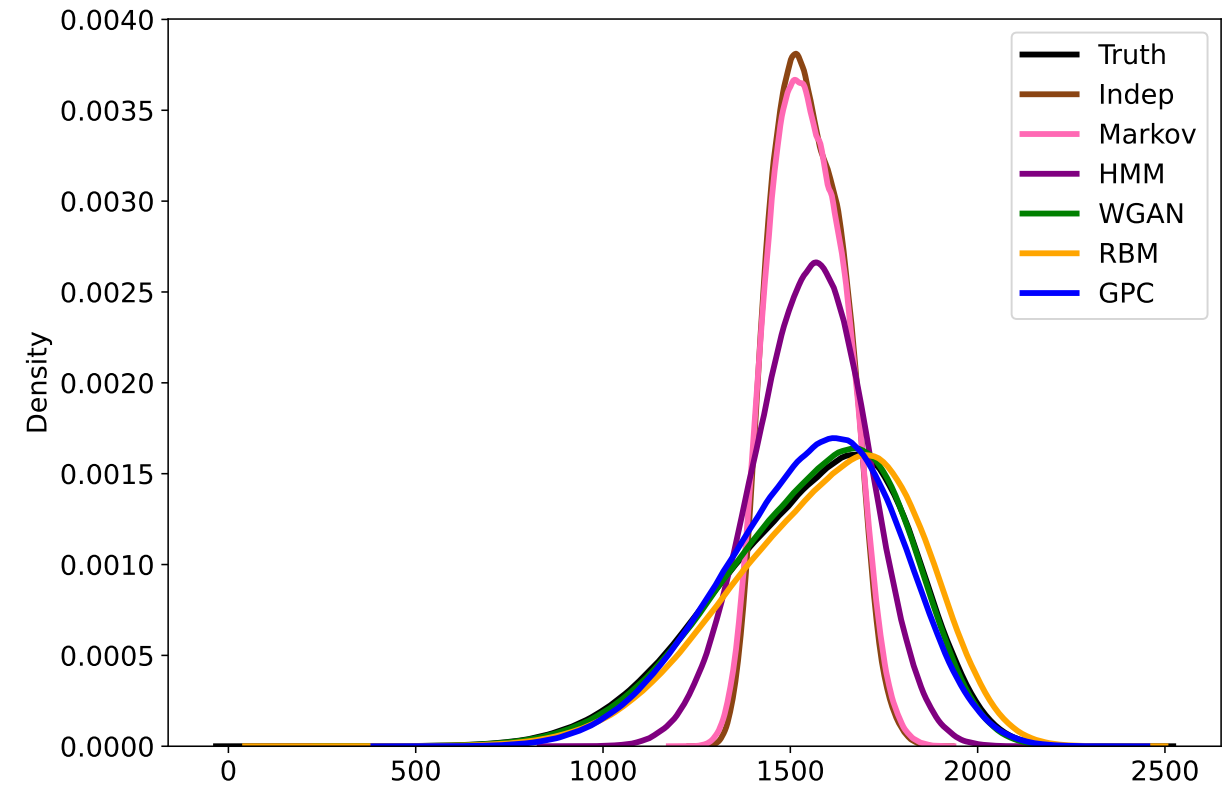

(b) Between datasets and test set (10K)

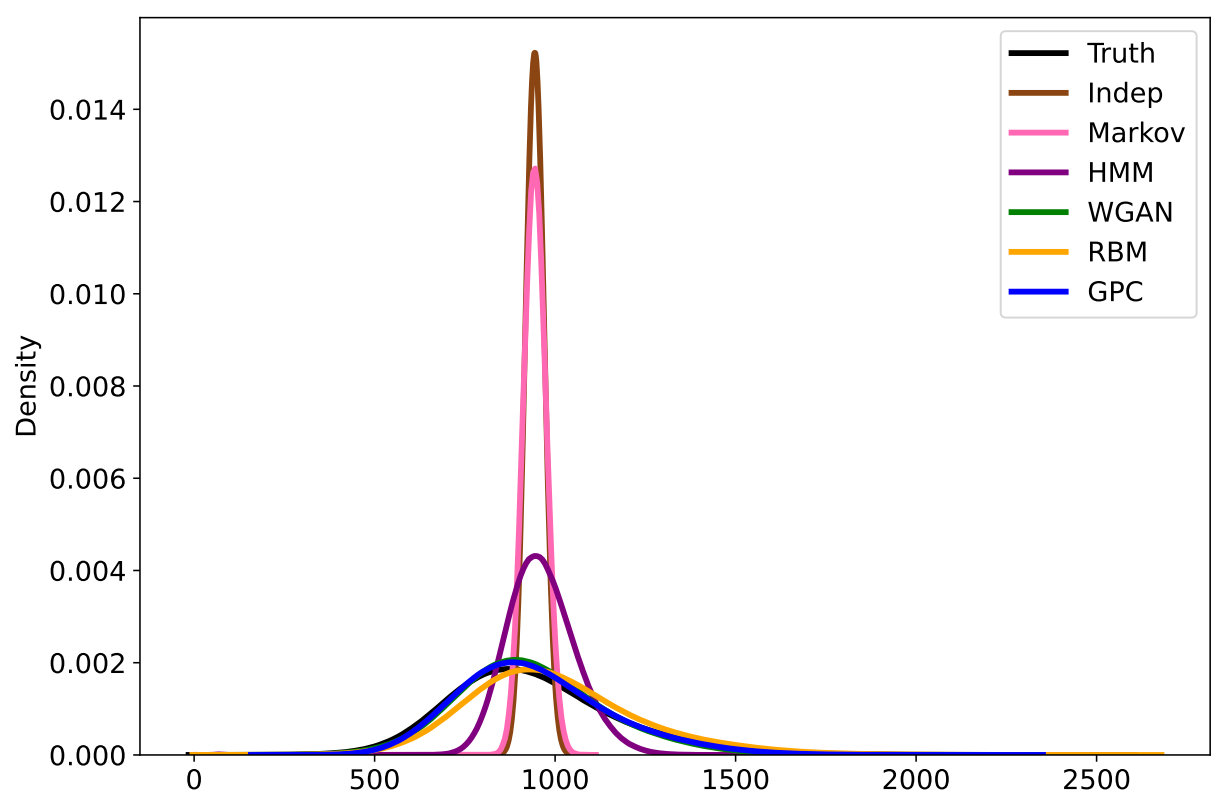

(c) Within each dataset (UKBB)

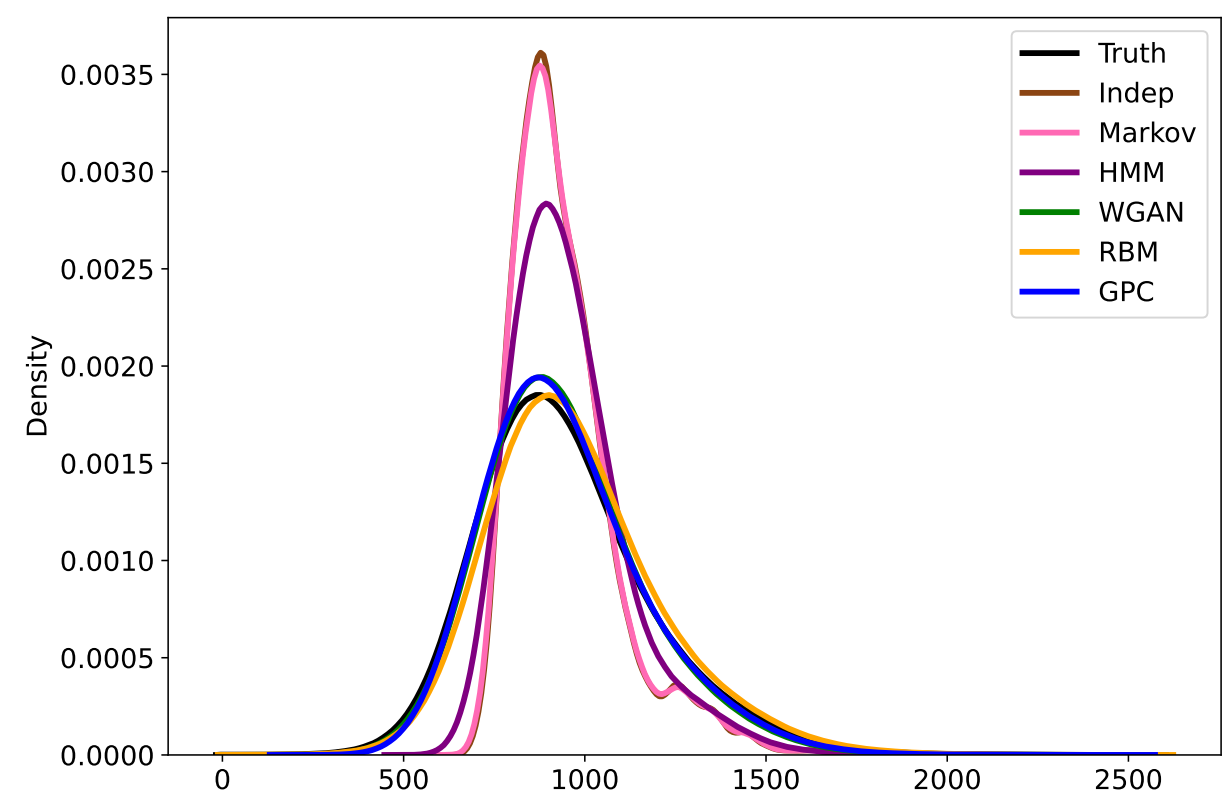

(d) Between datasets and test set (UKBB)

S1 Fig. **Distribution of haplotypic pairwise Euclidean distances.** (a–b) show results for the 1KG dataset; (c–d) for the UKBB dataset. (a,c) visualize distances *within* each dataset (i.e., intra-group variation). (b,d) show distances *between* the AG-generated samples and the test set (i.e., realism and mode collapse). Each panel compares across different generative models (see S1 Table for detailed metrics).
