# Supplementary material for: GPC: An expressive and tractable deep generative model for genetic variation data
Source: PLoS Genet. 2026 Oct 6;22(10):e1012321. doi: 10.1371/journal.pgen.1012321 (PMC13652338; doi:10.1371/journal.pgen.1012321)
Supplement: Supplementary file 2 [file pgen.1012321.s002.pdf]

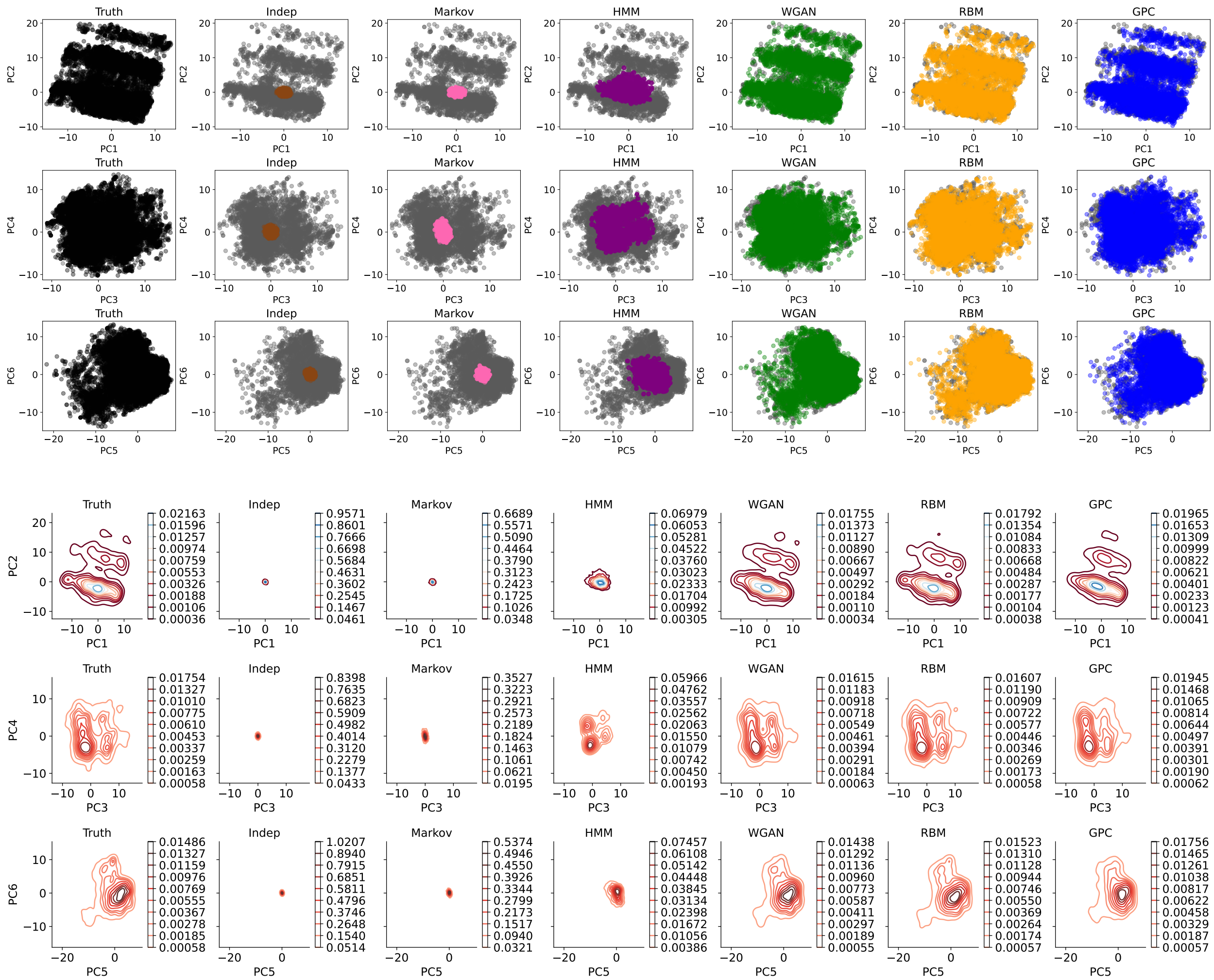

S2 Fig. **Principal components analysis for models trained on the UKBB dataset**. The top half shows a scatter plot of the top six principal components of the test set (gray) vs. AGs generated via Indep (brown), Markov (pink), HMM (purple), WGAN (green), RBM (orange), and GPC (blue). The left plot considers the train set as "perfectly" generated data (black). The bottom half of the figure shows a density map of the principal components for each dataset. All deep generative models are able to capture the population structure with good accuracy (see S2 Table for detailed metrics).
