# Supplementary material for: GPC: An expressive and tractable deep generative model for genetic variation data
Source: PLoS Genet. 2026 Oct 6;22(10):e1012321. doi: 10.1371/journal.pgen.1012321 (PMC13652338; doi:10.1371/journal.pgen.1012321)
Supplement: Supplementary file 3 [file pgen.1012321.s003.pdf]

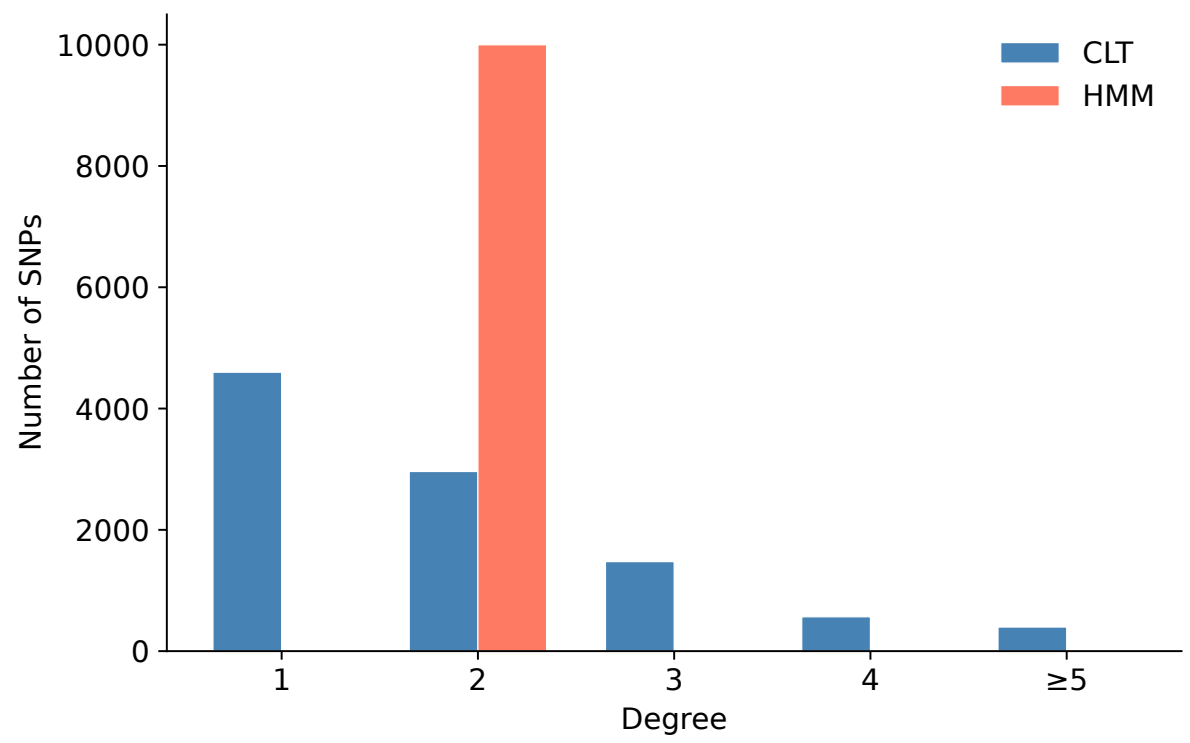

(a)

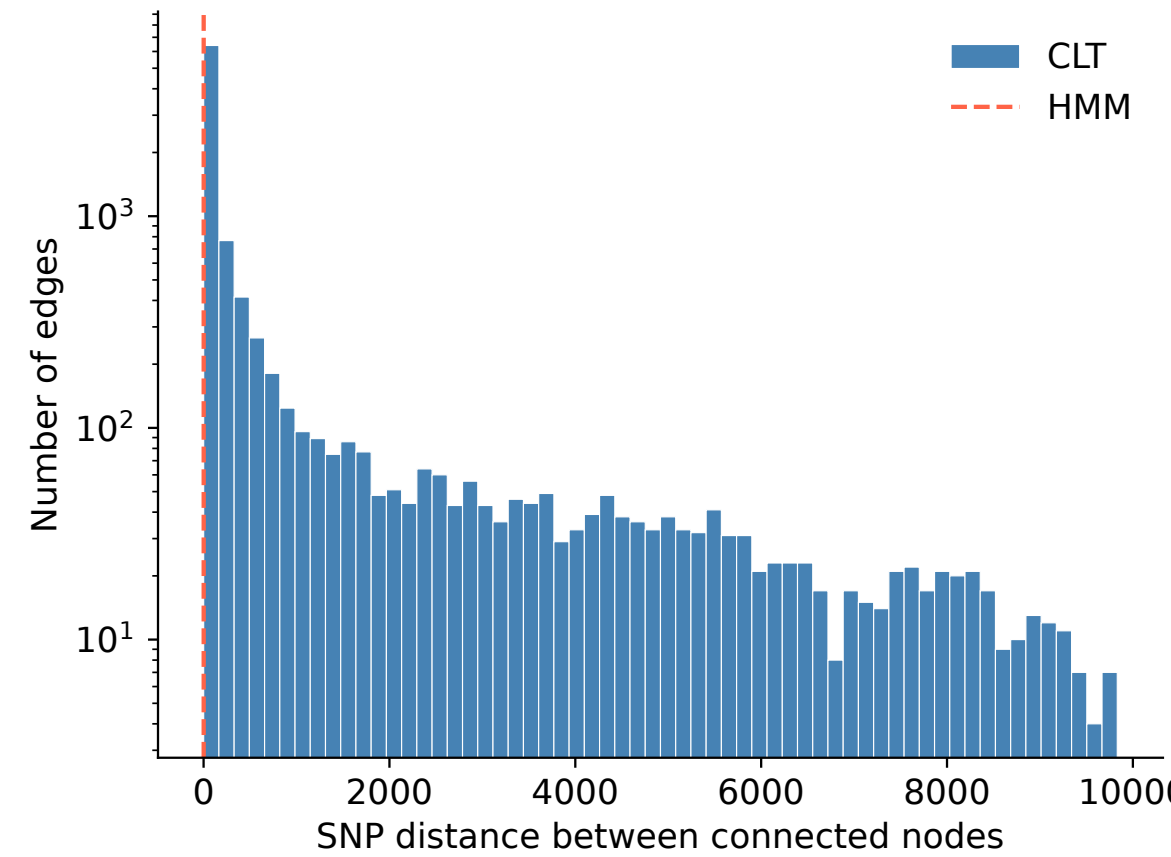

(b)

**Property**	**CLT**	**HMM**
Total nodes (SNPs)	10,000	10,000
Leaf nodes (degree 1)	4,599	2
Degree-2 nodes	2,962	9,998
High-degree nodes (degree ≥ 5)	397	0
Maximum node degree	161	2
Adjacent edges ($\|i-j\| = 1$)	542 (5.4%)	9,999 (100%)
Long-range edges ($\|i-j\| > 1{,}000$)	1,834 (18.3%)	0 (0%)
Maximum edge span ($\|i-j\|$)	9,832	1

(c)

S3 Fig. **Comparison of the structures of Chow–Liu Tree (CLT) and a Hidden Markov Model (HMM) on the 1KG dataset.** **(a)** Degree distribution of CLT nodes versus HMM nodes. In an HMM chain over N SNPs, exactly 2 nodes have degree 1 and all remaining $N-2$ nodes have degree 2. The CLT instead exhibits a highly heterogeneous degree distribution, with 4,599 leaf nodes, 2,962 degree-2 nodes, and 397 nodes with degree ≥ 5, including one node (SNP 7579) with maximum degree 161. **(b)** Distribution of sequential edge spans ($|i-j|$ for each connected pair of SNPs), plotted on a log-scale y-axis. An HMM consists entirely of adjacent-SNP edges (dashed red line at distance 1), whereas the CLT spans distances up to 9,832 SNP positions, with 18.3% of edges connecting SNPs more than 1,000 positions apart. **(c)** Summary statistics comparing CLT and HMM topologies. Together, these panels demonstrate that the Chow–Liu algorithm learns a tree structure fundamentally distinct from a chain, directly linking SNPs with strong mutual information regardless of their genomic proximity and thereby encoding both local and long-range LD structure.
