# Supplementary material for: GPC: An expressive and tractable deep generative model for genetic variation data
Source: PLoS Genet. 2026 Oct 6;22(10):e1012321. doi: 10.1371/journal.pgen.1012321 (PMC13652338; doi:10.1371/journal.pgen.1012321)
Supplement: Supplementary file 4 [file pgen.1012321.s004.pdf]

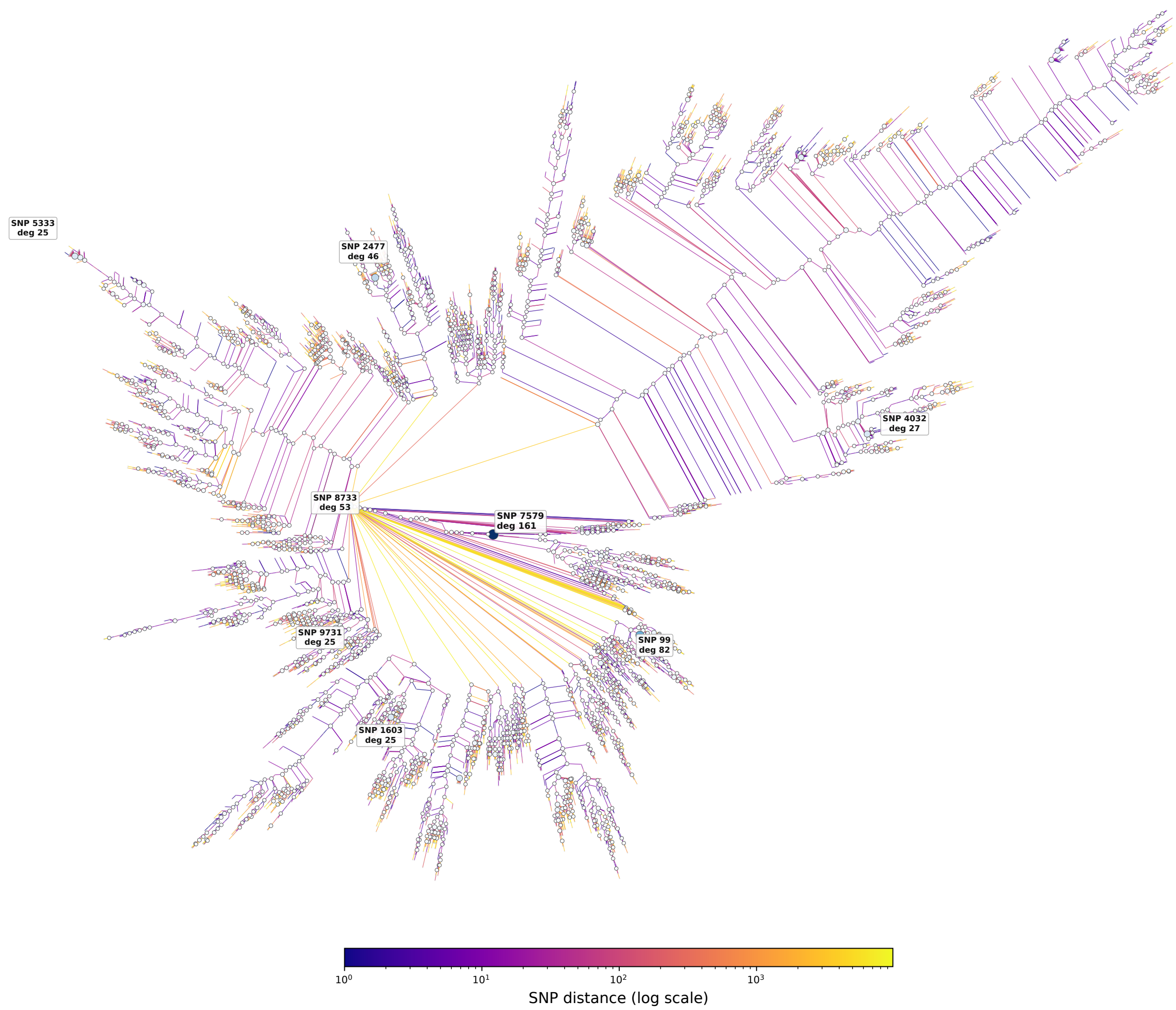

S4 Fig. **Radial visualization of the Chow–Liu Tree (CLT) learned from the 1KG dataset.** Each of the 10,000 nodes represents one SNP; edges connect pairs of SNPs whose latent variables are adjacent in the tree. Edge color encodes the sequential SNP distance $|i-j|$ on a log scale, ranging from deep purple (nearby SNPs, $|i-j| \approx 1$) to yellow (SNPs thousands of positions apart, $|i-j| \sim 10^3$). High-degree nodes (degree ≥ 3) are shown as filled circles scaled by degree; leaf and degree-2 nodes appear as small gray circles. The top 8 highest-degree nodes are labeled with their SNP index and degree. In contrast to an HMM, which would appear as a single unbranched chain, the CLT exhibits a highly branching structure. For the highest-degree node SNP 7579 (degree 161), the edges leaving it are not visually apparent as individual lines because the vast majority of its 161 connected SNPs are themselves leaves with no further subtrees extending from them; they appear simply as the small gray circles immediately surrounding it. This contrasts with SNP 8733 (degree 53), whose connected nodes have large subtrees branching out from them, producing the visually prominent clusters that extend outward from that region of the tree. The long orange and yellow edges encode direct connections between SNPs thousands of genomic positions apart, capturing long-range LD that a chain topology cannot represent without propagating information through all intermediate variables.
