# Supplementary material for: GPC: An expressive and tractable deep generative model for genetic variation data
Source: PLoS Genet. 2026 Oct 6;22(10):e1012321. doi: 10.1371/journal.pgen.1012321 (PMC13652338; doi:10.1371/journal.pgen.1012321)
Supplement: Supplementary file 5 [file pgen.1012321.s005.pdf]

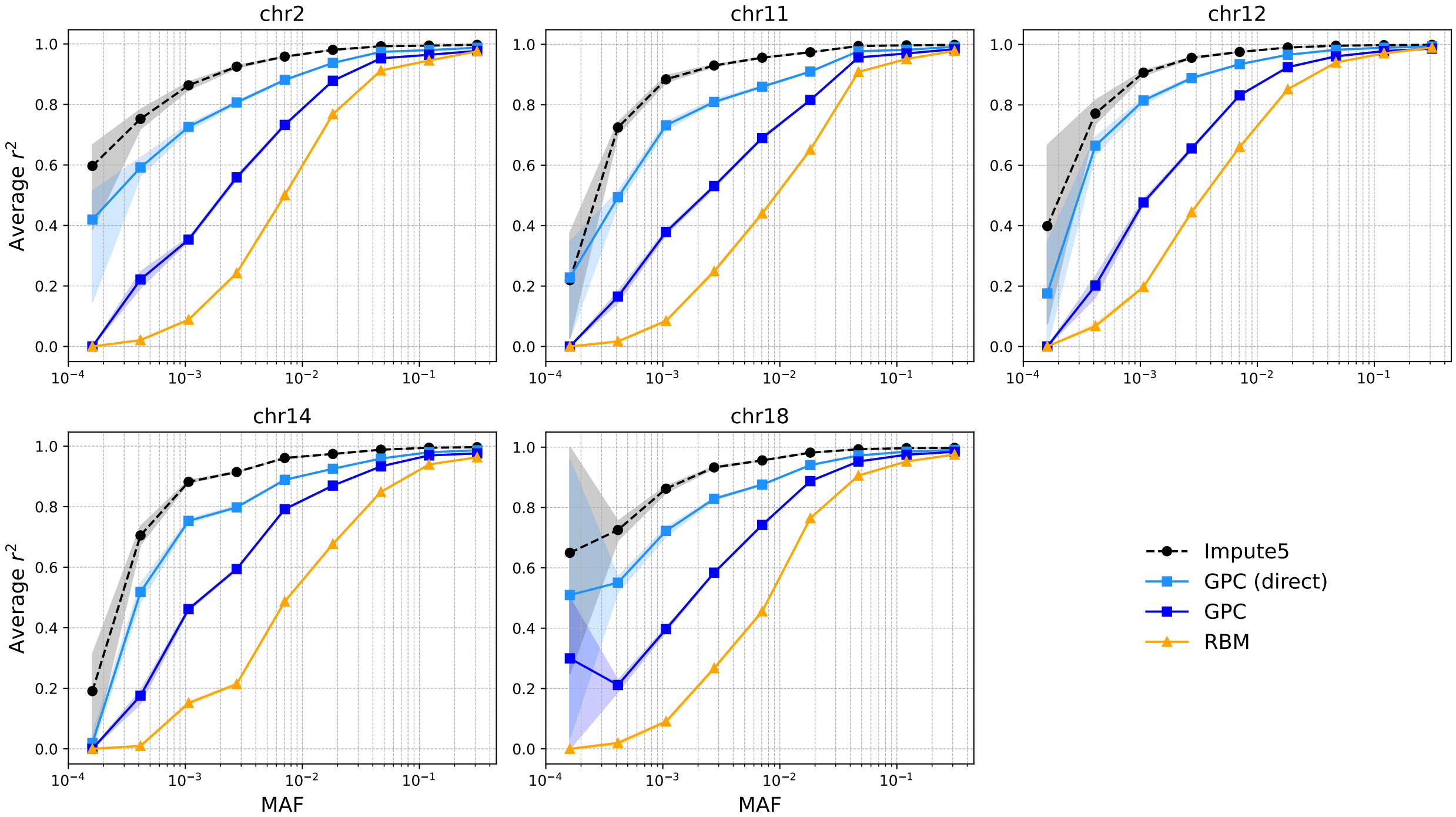

S5 Fig. **General imputation across additional UKBB regions.** The black and light blue lines denote imputation results using Impute5 with real reference genomes and direct imputation using GPC, respectively. The other lines show imputation results using Impute5 with AG reference panels. r^2 is shown as a function of MAF for each of the five additional chromosomes (chr2, 11, 12, 14, 18) separately (see S5 Table for detailed metrics).
