# Supplementary material for: GPC: An expressive and tractable deep generative model for genetic variation data
Source: PLoS Genet. 2026 Oct 6;22(10):e1012321. doi: 10.1371/journal.pgen.1012321 (PMC13652338; doi:10.1371/journal.pgen.1012321)
Supplement: Supplementary file 6 [file pgen.1012321.s006.pdf]

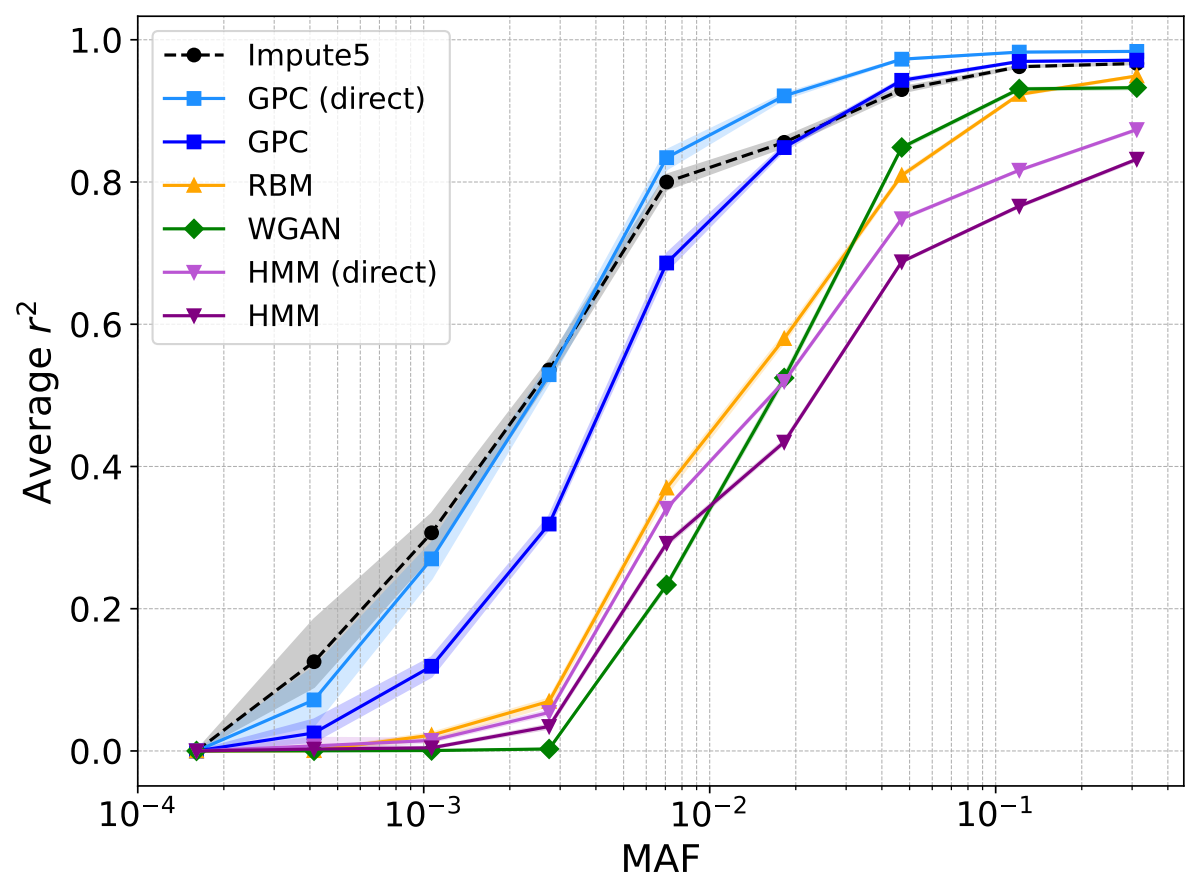

(a) Non-European target

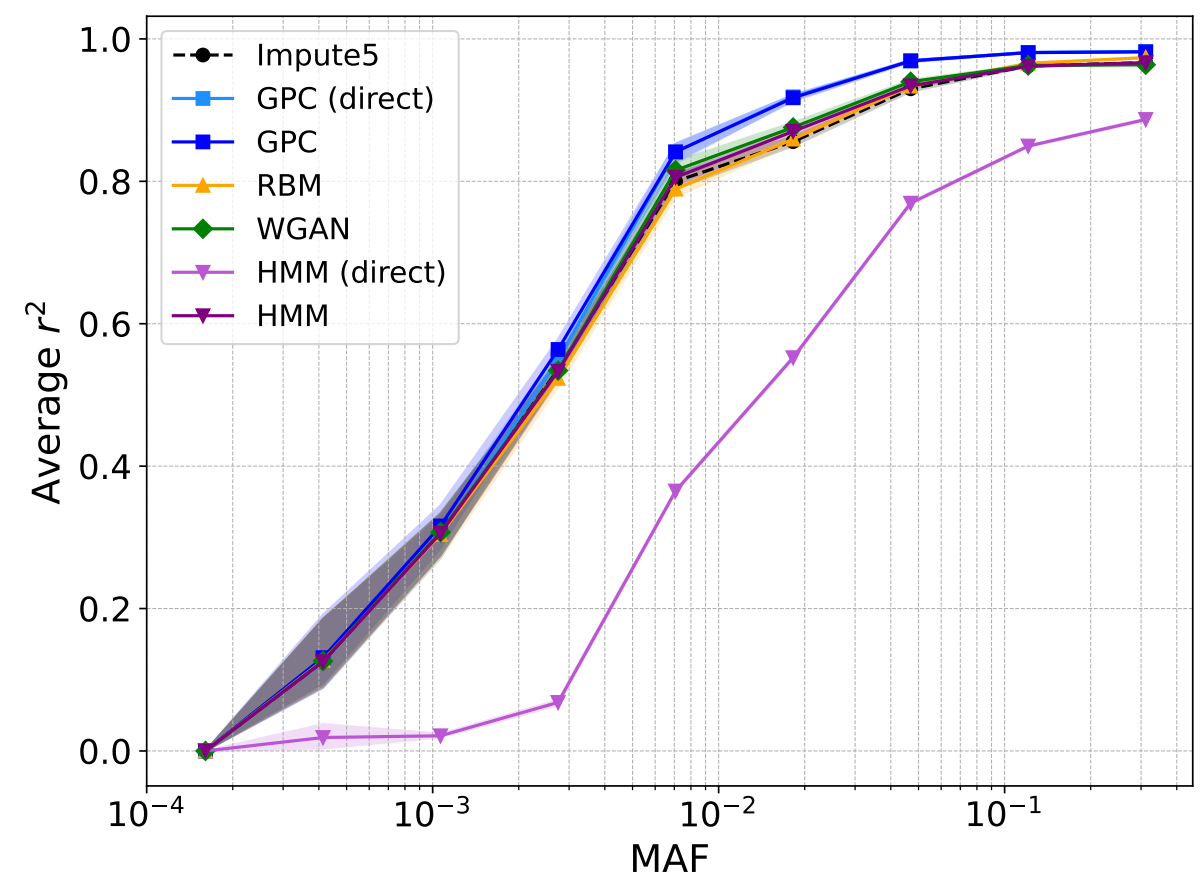

(b) Non-European target

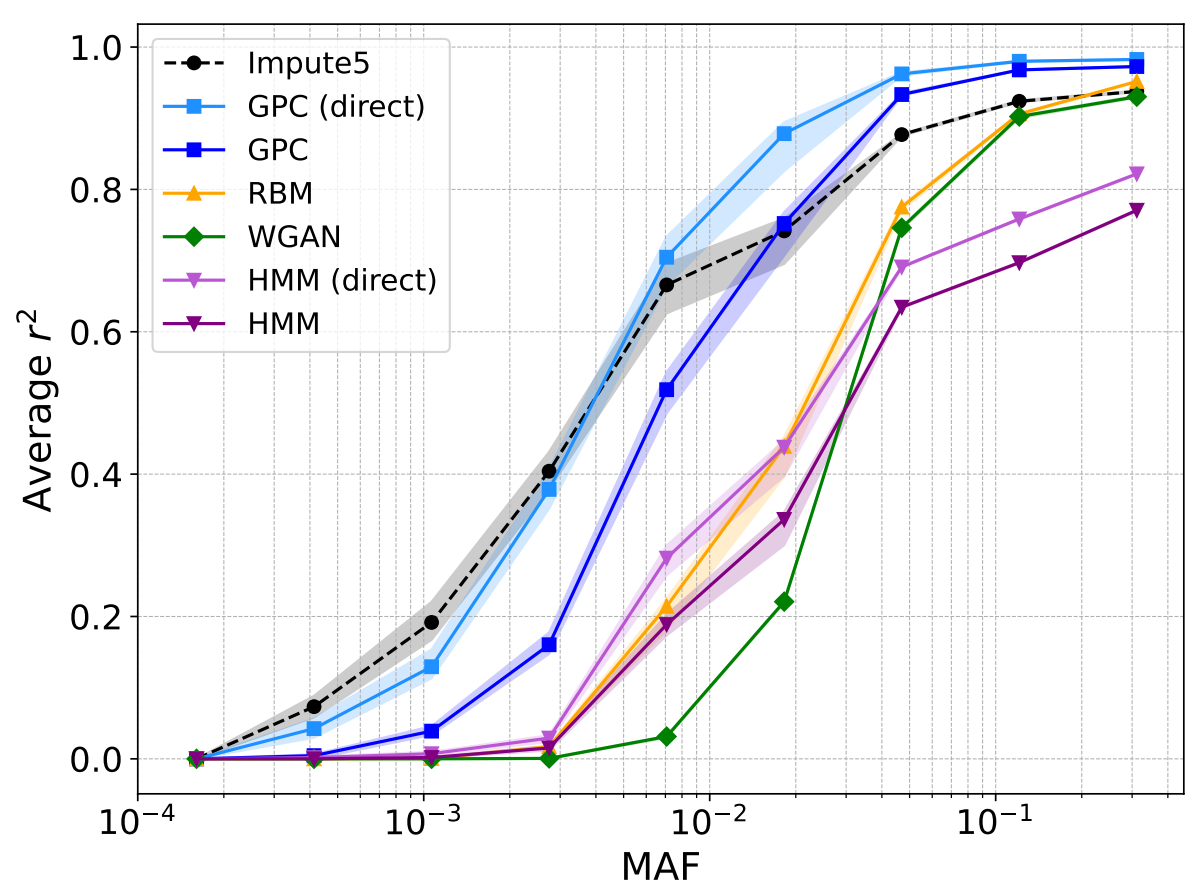

(c) African target

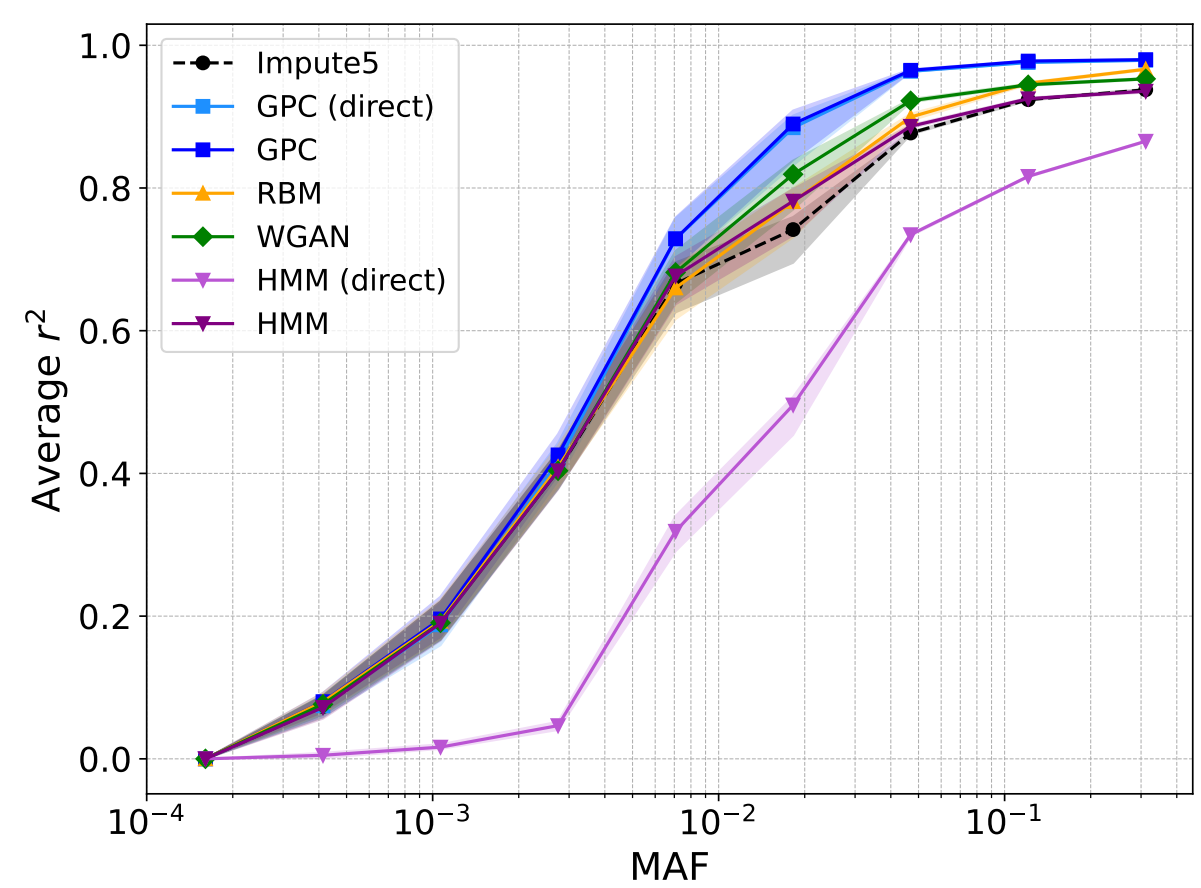

(d) African target

S6 Fig. **Population-specific imputation in the UKBB dataset.** On the left, the AG lines show results using population-specific AGs alone while the light blue line shows direct imputation with GPC trained on population-specific genomes (where the target genomes correspond to non-European populations in part a and African populations in part c). On the right, the AG lines show results using combined reference panels (real European data plus population-specific AGs), and the light blue line shows direct imputation with GPC trained on both European and population-specific genomes (where the target genomes correspond to non-European populations in part b and African populations in part d). The black line shows results using Impute5 with real European data as the reference panel (see S7 Table for detailed metrics).
