# Supplementary material for: GPC: An expressive and tractable deep generative model for genetic variation data
Source: PLoS Genet. 2026 Oct 6;22(10):e1012321. doi: 10.1371/journal.pgen.1012321 (PMC13652338; doi:10.1371/journal.pgen.1012321)
Supplement: Supplementary file 7 [file pgen.1012321.s007.pdf]

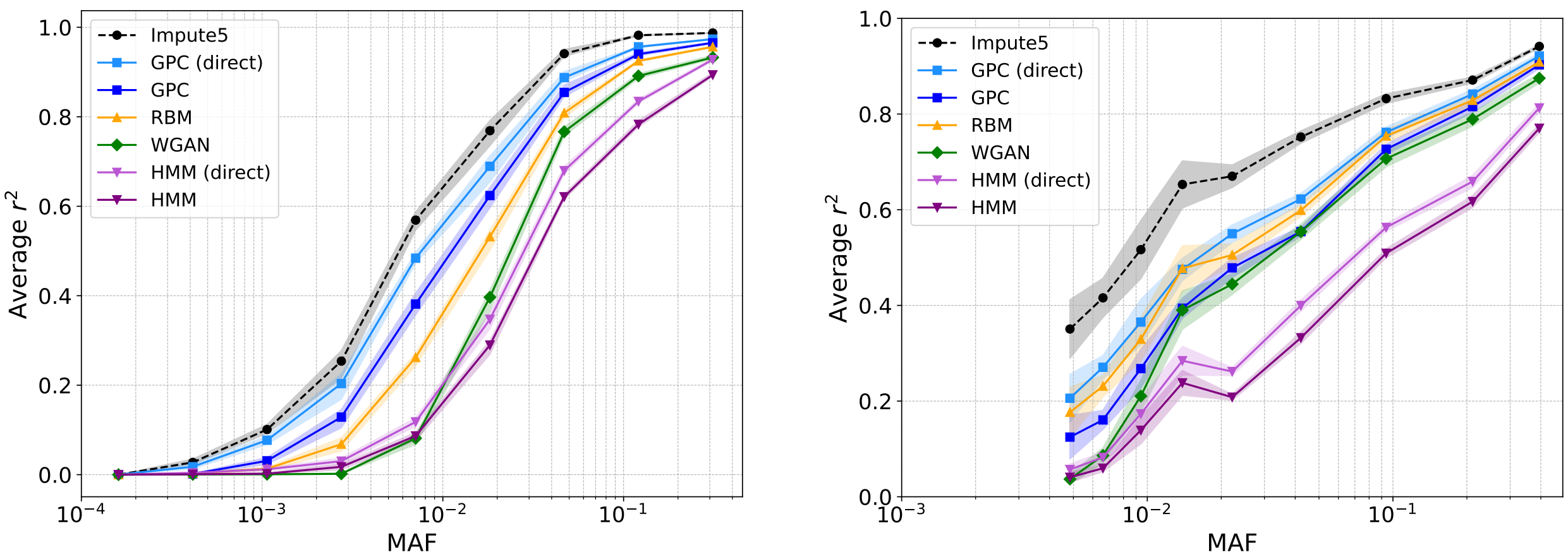

(a) 1KG

(b) High-coverage 1KG, array-based

S7 Fig. **Imputation accuracy in admixed individuals.** As Fig 6a and Fig 8, but with the target set restricted to the 212 admixed haplotypes (106 individuals) of the held-out split: the four American populations (CLM, MXL, PEL, PUR) and the two recently admixed African-ancestry populations (ASW, ACB). No model is retrained; these are the cosmopolitan models of the general 80/20 experiment, scored on a subset of their own test set. Every method is less accurate than on the full test set, as expected, but the ordering is unchanged and GPC (direct) remains the most accurate generative model in both settings. Detailed values are in S10 Table.
