# Supplementary material for: GPC: An expressive and tractable deep generative model for genetic variation data
Source: PLoS Genet. 2026 Oct 6;22(10):e1012321. doi: 10.1371/journal.pgen.1012321 (PMC13652338; doi:10.1371/journal.pgen.1012321)
Supplement: Supplementary file 8 [file pgen.1012321.s008.pdf]

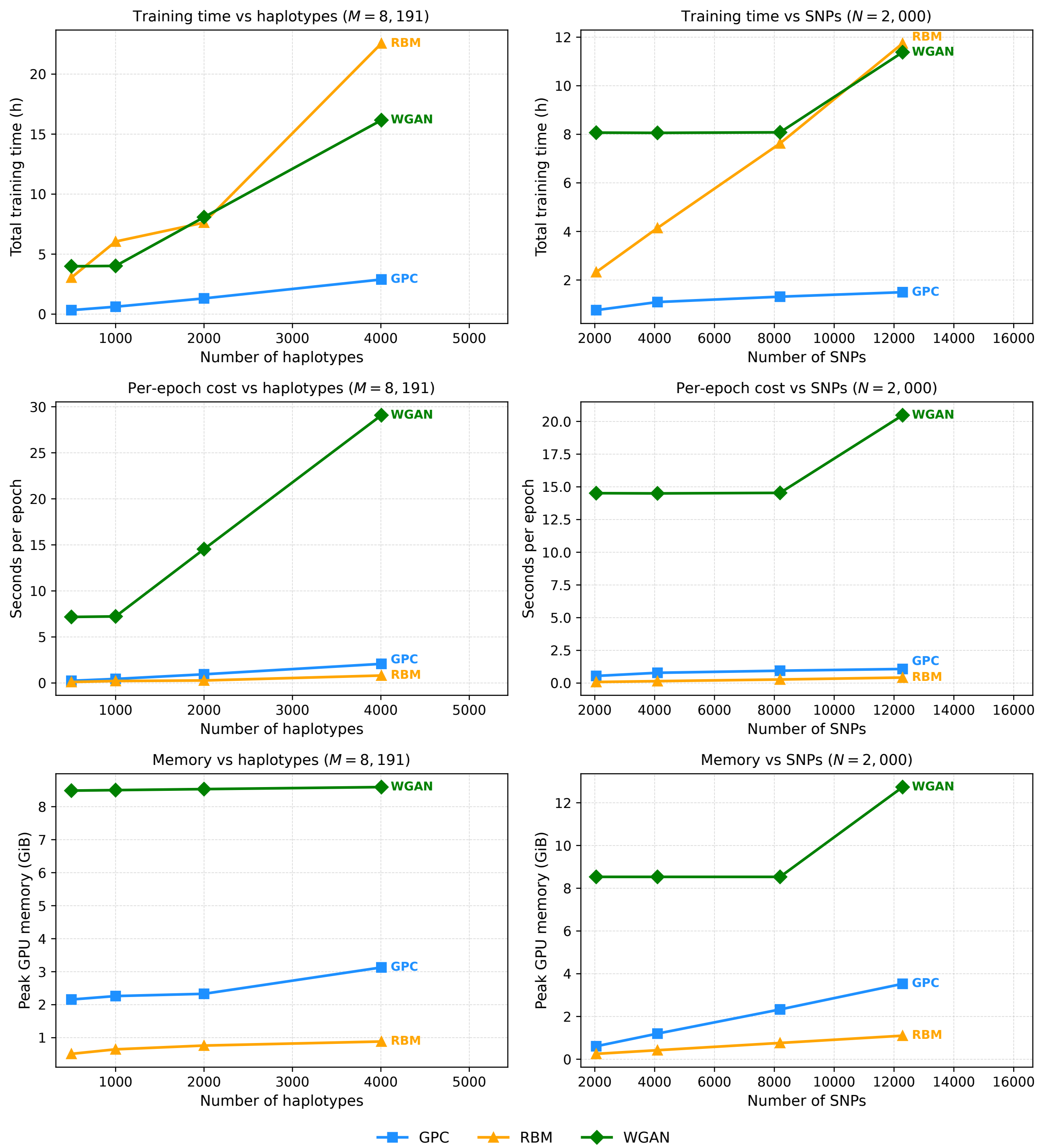

S8 Fig. **Training cost and peak memory against dataset size.** Measured on one NVIDIA RTX A5000 with no other process on the device, each method at the default parameters of its published training script. Left column varies haplotypes N, right column varies SNPs M; rows are total training time, per-epoch cost, and peak GPU memory. Total time is per-epoch cost times each method's epoch count (5,000 for GPC, 100,000 for RBM, 2,001 for WGAN), which is why GPC is fastest overall despite a more expensive epoch than RBM. WGAN cannot train on fewer than 8,191 SNPs, since its critic halves the sequence twelve times and a shorter input leaves a single position at the innermost layer; the two smaller sizes are zero-padded up to 8,191 as its published pipeline does, so its cost is flat below that.
