# Supplementary material for: GPC: An expressive and tractable deep generative model for genetic variation data
Source: PLoS Genet. 2026 Oct 6;22(10):e1012321. doi: 10.1371/journal.pgen.1012321 (PMC13652338; doi:10.1371/journal.pgen.1012321)
Supplement: Supplementary file 9 [file pgen.1012321.s009.pdf]

Dataset		Truth	Indep	Markov	HMM	WGAN	RBM	GPC
**1KG**	Within	10.66	180.23	172.70	105.88	**14.73**	61.99	23.59
	Between	5.34	128.24	125.61	86.25	**6.35**	31.50	18.73
	PCA1-2	0.0026	0.2355	0.2085	0.0348	0.0028	**0.0027**	0.0039
	PCA3-4	0.0022	0.1476	0.1148	0.0123	**0.0023**	0.0024	0.0028
	PCA5-6	0.0023	0.1011	0.0822	0.0042	0.0024	**0.0023**	0.0029
**UKBB**	Within	3.57	168.88	164.55	117.91	25.16	49.52	**20.86**
	Between	1.81	80.20	79.36	64.25	13.08	25.81	**11.88**
	PCA1-2	0.0019	0.0909	0.0846	0.0072	0.0022	**0.0021**	0.0024
	PCA3-4	0.0019	0.0656	0.0634	0.0244	0.0022	**0.0022**	0.0026
	PCA5-6	0.0019	0.0634	0.0542	0.0117	0.0021	**0.0020**	0.0023

S1 Table. **Distance metrics between real and generated data. Within, Between** (S1 Fig): Within denotes the Wasserstein distance between the distributions of pairwise haplotypic distances computed within a single dataset (e.g., generated–generated) and the corresponding distribution computed within the real test set (real–real). Between denotes the Wasserstein distance between the distribution of pairwise haplotypic distances computed between generated and real test individuals (generated–real) and the distribution computed within the real test set (real–real). In both cases, the real test set serves as the reference distribution. **PCA1-2, PCA3-4, PCA5-6**: Wasserstein 2D distances between the PCA representations of real (test set) versus generated individuals. Truth represents the training set. For each PCA distance, one PCA is fit on the method AGs and test data. Bolded values indicate the best among all compared models. Because a separate PCA is fit for each method, these PCA distances are a within-method diagnostic and are not directly comparable across methods; S2 Table reports the same distances in a single common basis shared by all methods.
