# Supplementary material for: GPC: An expressive and tractable deep generative model for genetic variation data
Source: PLoS Genet. 2026 Oct 6;22(10):e1012321. doi: 10.1371/journal.pgen.1012321 (PMC13652338; doi:10.1371/journal.pgen.1012321)
Supplement: Supplementary file 10 [file pgen.1012321.s010.pdf]

Dataset		Truth	Indep	Markov	HMM	WGAN	RBM	GPC
**1KG**	PCA1-2	0.0026	0.2491	0.2260	0.0840	0.0029	**0.0026**	0.0059
	PCA3-4	0.0022	0.1402	0.1139	0.0129	0.0023	**0.0023**	0.0036
	PCA5-6	0.0022	0.1072	0.0855	0.0144	0.0024	**0.0023**	0.0024
**UKBB**	PCA1-2	0.0018	0.0856	0.0816	0.0390	0.0022	**0.0020**	0.0026
	PCA3-4	0.0020	0.0857	0.0720	0.0151	0.0023	**0.0020**	0.0024
	PCA5-6	0.0019	0.0658	0.0593	0.0273	0.0022	**0.0022**	0.0025

S2 Table. **PCA distances in a common basis.** Wasserstein 2D distances between the PCA representations of real (test set) versus generated individuals, computed after fitting a single PCA once on the union of all eight datasets (the training set, the test set and the six sets of artificial genomes) and projecting every dataset into that same space. This is the space shown in Figs 3 and S2. Unlike S1 Table, where a separate PCA is fit for each method, these values share one set of axes and one scaling and are therefore directly comparable across methods. Truth represents the training set. Bolded values indicate the best among all compared models. The chain-structured HMM is the setting most affected by the change of basis (PCA1-2: $0.0348 \rightarrow 0.0840$ on 1KG and $0.0072 \rightarrow 0.0390$ on UKBB), because a per-method fit is free to orient its axes around that method's artificial genomes.
