# Supplementary material for: GPC: An expressive and tractable deep generative model for genetic variation data
Source: PLoS Genet. 2026 Oct 6;22(10):e1012321. doi: 10.1371/journal.pgen.1012321 (PMC13652338; doi:10.1371/journal.pgen.1012321)
Supplement: Supplementary file 11 [file pgen.1012321.s011.pdf]

**Dataset**	**Metric**	Truth	Indep	Markov	HMM	WGAN	RBM	GPC
**1KG**	MAE	0.0008	0.1676	0.0708	0.0153	0.0237	0.0182	**0.0053**
	RMSE	0.0011	0.2101	0.1031	0.0240	0.0361	0.0307	**0.0056**
**UKBB**	MAE	0.0003	0.1111	0.0481	0.0153	0.0186	0.0295	**0.0047**
	RMSE	0.0004	0.1336	0.0649	0.0262	0.0283	0.0414	**0.0052**

S3 Table. **LD decay error metrics for Fig 4**. Mean absolute error (MAE) and root mean squared error (RMSE) are computed as differences between the mean values of each bin on the LD decay curves across genomic distance. Lower values indicate better preservation of LD structure; best-performing values are bolded.
