# Supplementary material for: GPC: An expressive and tractable deep generative model for genetic variation data
Source: PLoS Genet. 2026 Oct 6;22(10):e1012321. doi: 10.1371/journal.pgen.1012321 (PMC13652338; doi:10.1371/journal.pgen.1012321)
Supplement: Supplementary file 12 [file pgen.1012321.s012.pdf]

1KG

Method	Mean r^2 [95% CI]
**All SNPs**	
Impute5	0.649 [0.643, 0.655]
RBM (full)	0.510 [0.506, 0.514]
WGAN (full)	0.428 [0.426, 0.430]
RBM	0.494 [0.491, 0.496]
WGAN	0.445 [0.443, 0.447]
HMM	0.409 [0.406, 0.413]
HMM (direct)	0.441 [0.437, 0.444]
GPC	0.540 [0.536, 0.544]
**GPC (direct)**	**0.593 [0.589, 0.598]**
**Low-freq** (MAF < 1%)	
Impute5	0.298 [0.286, 0.310]
RBM (full)	0.095 [0.088, 0.103]
WGAN (full)	0.004 [0.004, 0.005]
RBM	0.066 [0.060, 0.072]
WGAN	0.009 [0.009, 0.010]
HMM	0.024 [0.018, 0.030]
HMM (direct)	0.039 [0.033, 0.045]
GPC	0.132 [0.123, 0.140]
**GPC (direct)**	**0.218 [0.208, 0.228]**
**Rare** (MAF < 0.1%)	
Impute5	0.089 [0.078, 0.099]
RBM (full)	0.012 [0.007, 0.017]
WGAN (full)	0.000 [0.000, 0.000]
RBM	0.007 [0.005, 0.009]
WGAN	0.001 [0.000, 0.001]
HMM	0.002 [0.001, 0.004]
HMM (direct)	0.006 [0.004, 0.009]
GPC	0.012 [0.008, 0.017]
**GPC (direct)**	**0.053 [0.044, 0.061]**

UKBB

Method	Mean r^2 [95% CI]
**All SNPs**	
Impute5	0.957 [0.954, 0.960]
RBM	0.669 [0.667, 0.670]
WGAN	0.523 [0.522, 0.524]
HMM	0.414 [0.413, 0.415]
HMM (direct)	0.468 [0.467, 0.469]
GPC	0.750 [0.748, 0.751]
**GPC (direct)**	**0.906 [0.903, 0.909]**
**Low-freq** (MAF < 1%)	
Impute5	0.914 [0.907, 0.922]
RBM	0.381 [0.377, 0.384]
WGAN	0.087 [0.086, 0.088]
HMM	0.099 [0.098, 0.100]
HMM (direct)	0.138 [0.137, 0.139]
GPC	0.525 [0.522, 0.528]
**GPC (direct)**	**0.828 [0.822, 0.835]**
**Rare** (MAF < 0.1%)	
Impute5	0.818 [0.796, 0.839]
RBM	0.118 [0.112, 0.123]
WGAN	0.000 [0.000, 0.000]
HMM	0.010 [0.007, 0.013]
HMM (direct)	0.039 [0.037, 0.042]
GPC	0.263 [0.252, 0.274]
**GPC (direct)**	**0.709 [0.687, 0.730]**

S4 Table. **Summarized metrics for Fig 6.** Mean imputation performance across 10 bootstrapped replicates for all, low-frequency (MAF < 1%), and rare (MAF < 0.1%) SNPs. Best generative model is bolded.
