# Supplementary material for: GPC: An expressive and tractable deep generative model for genetic variation data
Source: PLoS Genet. 2026 Oct 6;22(10):e1012321. doi: 10.1371/journal.pgen.1012321 (PMC13652338; doi:10.1371/journal.pgen.1012321)
Supplement: Supplementary file 13 [file pgen.1012321.s013.pdf]

Chromosome 2

Method	Mean r^2 [95% CI]
**All SNPs**	
Impute5	0.949 [0.945, 0.952]
RBM	0.605 [0.603, 0.606]
GPC	0.746 [0.744, 0.748]
**GPC (direct)**	**0.884 [0.882, 0.887]**
**Low-freq** (MAF $< 1\%$)	
Impute5	0.894 [0.886, 0.902]
RBM	0.222 [0.219, 0.225]
GPC	0.491 [0.486, 0.496]
**GPC (direct)**	**0.774 [0.768, 0.780]**
**Rare** (MAF $< 0.1\%$)	
Impute5	0.805 [0.782, 0.827]
RBM	0.043 [0.037, 0.048]
GPC	0.269 [0.253, 0.286]
**GPC (direct)**	**0.654 [0.635, 0.673]**

Chromosome 11

Method	Mean r^2 [95% CI]
**All SNPs**	
Impute5	0.959 [0.956, 0.962]
RBM	0.640 [0.639, 0.642]
GPC	0.770 [0.768, 0.772]
**GPC (direct)**	**0.895 [0.891, 0.898]**
**Low-freq** (MAF $< 1\%$)	
Impute5	0.903 [0.896, 0.911]
RBM	0.214 [0.211, 0.218]
GPC	0.484 [0.478, 0.490]
**GPC (direct)**	**0.769 [0.760, 0.778]**
**Rare** (MAF $< 0.1\%$)	
Impute5	0.801 [0.778, 0.824]
RBM	0.033 [0.030, 0.035]
GPC	0.272 [0.257, 0.287]
**GPC (direct)**	**0.621 [0.598, 0.644]**

Chromosome 12

Method	Mean r^2 [95% CI]
**All SNPs**	
Impute5	0.970 [0.968, 0.973]
RBM	0.717 [0.715, 0.718]
GPC	0.823 [0.821, 0.824]
**GPC (direct)**	**0.934 [0.931, 0.937]**
**Low-freq** (MAF $< 1\%$)	
Impute5	0.937 [0.932, 0.942]
RBM	0.423 [0.419, 0.426]
GPC	0.636 [0.632, 0.639]
**GPC (direct)**	**0.869 [0.863, 0.876]**
**Rare** (MAF $< 0.1\%$)	
Impute5	0.823 [0.795, 0.851]
RBM	0.115 [0.105, 0.126]
GPC	0.330 [0.314, 0.347]
**GPC (direct)**	**0.723 [0.696, 0.750]**

Chromosome 14

Method	Mean r^2 [95% CI]
**All SNPs**	
Impute5	0.946 [0.943, 0.948]
RBM	0.569 [0.567, 0.571]
GPC	0.761 [0.759, 0.763]
**GPC (direct)**	**0.878 [0.874, 0.881]**
**Low-freq** (MAF $< 1\%$)	
Impute5	0.897 [0.891, 0.903]
RBM	0.243 [0.240, 0.247]
GPC	0.561 [0.557, 0.566]
**GPC (direct)**	**0.781 [0.774, 0.788]**
**Rare** (MAF $< 0.1\%$)	
Impute5	0.790 [0.765, 0.814]
RBM	0.053 [0.047, 0.058]
GPC	0.293 [0.278, 0.308]
**GPC (direct)**	**0.627 [0.603, 0.651]**

Chromosome 18

Method	Mean r^2 [95% CI]
**All SNPs**	
Impute5	0.950 [0.947, 0.953]
RBM	0.598 [0.596, 0.599]
GPC	0.759 [0.757, 0.762]
**GPC (direct)**	**0.886 [0.883, 0.890]**
**Low-freq** (MAF $< 1\%$)	
Impute5	0.897 [0.890, 0.904]
RBM	0.222 [0.218, 0.225]
GPC	0.523 [0.517, 0.528]
**GPC (direct)**	**0.779 [0.773, 0.786]**
**Rare** (MAF $< 0.1\%$)	
Impute5	0.786 [0.759, 0.813]
RBM	0.037 [0.029, 0.044]
GPC	0.293 [0.276, 0.310]
**GPC (direct)**	**0.631 [0.605, 0.657]**

S5 Table. **Summarized metrics for S5 Fig (additional UKBB regions).** Mean imputation performance across 10 bootstrapped replicates for all, low-frequency (MAF $< 1\%$), and rare (MAF $< 0.1\%$) SNPs on five additional LD blocks in the UKBB dataset. Best generative model is bolded.
