# Supplementary material for: GPC: An expressive and tractable deep generative model for genetic variation data
Source: PLoS Genet. 2026 Oct 6;22(10):e1012321. doi: 10.1371/journal.pgen.1012321 (PMC13652338; doi:10.1371/journal.pgen.1012321)
Supplement: Supplementary file 14 [file pgen.1012321.s014.pdf]

Non-European

Method	Mean r^2 [95% CI]
**All SNPs**	
Impute5 (EUR)	0.485 [0.482, 0.488]
RBM	0.476 [0.474, 0.478]
WGAN	0.422 [0.421, 0.424]
HMM	0.412 [0.410, 0.413]
HMM (direct)	0.441 [0.440, 0.442]
GPC	0.522 [0.520, 0.524]
GPC (direct)†	0.563 [0.561, 0.564]†
RBM (combined)	0.528 [0.524, 0.532]
WGAN (combined)	0.507 [0.504, 0.510]
HMM (combined)	0.508 [0.505, 0.511]
HMM (direct combined)	0.444 [0.442, 0.445]
GPC (combined)	0.560 [0.555, 0.562]
**GPC (direct combined)**	**0.570 [0.567, 0.573]**
**Low-freq** (MAF $<$ 1%)	
Impute5 (EUR)	0.106 [0.101, 0.112]
RBM	0.042 [0.039, 0.046]
WGAN	0.003 [0.002, 0.004]
HMM	0.021 [0.019, 0.023]
HMM (direct)	0.037 [0.033, 0.040]
GPC	0.098 [0.095, 0.101]
GPC (direct)†	0.158 [0.156, 0.161]†
RBM (combined)	0.123 [0.116, 0.129]
WGAN (combined)	0.111 [0.105, 0.117]
HMM (combined)	0.118 [0.113, 0.124]
HMM (direct combined)	0.036 [0.033, 0.039]
GPC (combined)	0.160 [0.155, 0.165]
**GPC (direct combined)**	**0.174 [0.169, 0.178]**
**Rare** (MAF $<$ 0.1%)	
Impute5 (EUR)	0.021 [0.016, 0.025]
RBM	0.003 [0.001, 0.004]
WGAN	0.000 [0.000, 0.001]
HMM	0.001 [0.000, 0.002]
HMM (direct)	0.003 [0.002, 0.005]
GPC	0.004 [0.002, 0.006]
GPC (direct)†	0.021 [0.016, 0.026]†
RBM (combined)	0.024 [0.019, 0.029]
WGAN (combined)	0.021 [0.016, 0.025]
HMM (combined)	0.021 [0.017, 0.026]
HMM (direct combined)	0.004 [0.002, 0.006]
GPC (combined)	0.026 [0.021, 0.030]
**GPC (direct combined)**	**0.031 [0.025, 0.036]**

African

Method	Mean r^2 [95% CI]
**All SNPs**	
Impute5 (EUR)	0.368 [0.357, 0.379]
RBM	0.429 [0.424, 0.434]
WGAN	0.381 [0.379, 0.382]
HMM	0.343 [0.341, 0.344]
HMM (direct)	0.364 [0.361, 0.368]
GPC	0.442 [0.438, 0.446]
GPC (direct)†	0.462 [0.456, 0.468]†
RBM (combined)	0.446 [0.435, 0.456]
WGAN (combined)	0.423 [0.412, 0.435]
HMM (combined)	0.401 [0.390, 0.411]
HMM (direct combined)	0.390 [0.385, 0.395]
GPC (combined)	0.464 [0.454, 0.474]
**GPC (direct combined)**	**0.471 [0.461, 0.480]**
**Low-freq** (MAF $<$ 1%)	
Impute5 (EUR)	0.019 [0.013, 0.025]
RBM	0.028 [0.025, 0.030]
WGAN	0.009 [0.008, 0.009]
HMM	0.010 [0.009, 0.011]
HMM (direct)	0.012 [0.012, 0.013]
GPC	0.027 [0.025, 0.028]
GPC (direct)†	0.043 [0.040, 0.046]†
RBM (combined)	0.040 [0.033, 0.047]
WGAN (combined)	0.028 [0.022, 0.035]
HMM (combined)	0.027 [0.021, 0.034]
HMM (direct combined)	0.014 [0.013, 0.016]
GPC (combined)	0.043 [0.037, 0.050]
**GPC (direct combined)**	**0.053 [0.046, 0.059]**
**Rare** (MAF $<$ 0.1%)	
Impute5 (EUR)	0.003 [0.001, 0.005]
RBM	0.002 [0.001, 0.003]
WGAN	0.000 [0.000, 0.001]
HMM	0.001 [0.001, 0.002]
HMM (direct)	0.002 [0.001, 0.003]
GPC	0.001 [0.001, 0.001]
GPC (direct)†	0.006 [0.005, 0.008]†
RBM (combined)	0.004 [0.002, 0.006]
WGAN (combined)	0.003 [0.001, 0.005]
HMM (combined)	0.004 [0.001, 0.006]
HMM (direct combined)	0.002 [0.001, 0.003]
GPC (combined)	0.004 [0.002, 0.005]
**GPC (direct combined)**	**0.007 [0.005, 0.009]**

S6 Table. **Summarized metrics for Fig 7 (1KG).** Mean imputation performance across 10 bootstrapped replicates for all, low-frequency (MAF $<$ 1%), and rare (MAF $<$ 0.1%) SNPs. Best generative model using combined data is bolded; best using population-specific data only is marked with †.
