# Supplementary material for: GPC: An expressive and tractable deep generative model for genetic variation data
Source: PLoS Genet. 2026 Oct 6;22(10):e1012321. doi: 10.1371/journal.pgen.1012321 (PMC13652338; doi:10.1371/journal.pgen.1012321)
Supplement: Supplementary file 15 [file pgen.1012321.s015.pdf]

Non-European

Method	Mean r^2 [95% CI]
**All SNPs**	
Impute5 (EUR)	0.733 [0.725, 0.742]
RBM	0.495 [0.493, 0.497]
WGAN	0.461 [0.460, 0.463]
HMM	0.405 [0.404, 0.406]
HMM (direct)	0.448 [0.446, 0.449]
GPC	0.656 [0.651, 0.661]
GPC (direct)†	0.749 [0.742, 0.757]†
RBM (combined)	0.732 [0.723, 0.740]
WGAN (combined)	0.739 [0.731, 0.748]
HMM (combined)	0.736 [0.728, 0.744]
HMM (direct combined)	0.468 [0.466, 0.470]
**GPC (combined)**	**0.763 [0.755, 0.771]**
GPC (direct combined)	0.759 [0.752, 0.767]
**Low-freq** (MAF $<$ 1%)	
Impute5 (EUR)	0.506 [0.491, 0.522]
RBM	0.128 [0.123, 0.132]
WGAN	0.059 [0.058, 0.061]
HMM	0.090 [0.088, 0.092]
HMM (direct)	0.114 [0.112, 0.117]
GPC	0.333 [0.323, 0.343]
GPC (direct)†	0.498 [0.483, 0.513]†
RBM (combined)	0.498 [0.482, 0.514]
WGAN (combined)	0.510 [0.494, 0.526]
HMM (combined)	0.506 [0.491, 0.522]
HMM (direct combined)	0.129 [0.125, 0.133]
**GPC (combined)**	**0.530 [0.515, 0.546]**
GPC (direct combined)	0.522 [0.507, 0.537]
**Rare** (MAF $<$ 0.1%)	
Impute5 (EUR)	0.211 [0.174, 0.248]
RBM	0.004 [0.003, 0.006]
WGAN	0.000 [0.000, 0.000]
HMM	0.002 [-0.001, 0.004]
HMM (direct)	0.006 [0.000, 0.012]
GPC	0.054 [0.034, 0.073]
GPC (direct)†	0.159 [0.127, 0.190]†
RBM (combined)	0.210 [0.171, 0.248]
WGAN (combined)	0.209 [0.173, 0.245]
HMM (combined)	0.210 [0.173, 0.247]
HMM (direct combined)	0.016 [0.006, 0.026]
**GPC (combined)**	**0.217 [0.180, 0.255]**
GPC (direct combined)	0.210 [0.175, 0.246]

African

Method	Mean r^2 [95% CI]
**All SNPs**	
Impute5 (EUR)	0.645 [0.627, 0.663]
RBM	0.437 [0.428, 0.446]
WGAN	0.375 [0.374, 0.376]
HMM	0.350 [0.345, 0.355]
HMM (direct)	0.402 [0.395, 0.409]
GPC	0.582 [0.571, 0.593]
GPC (direct)†	0.679 [0.663, 0.694]†
RBM (combined)	0.659 [0.641, 0.678]
WGAN (combined)	0.668 [0.650, 0.686]
HMM (combined)	0.652 [0.635, 0.670]
HMM (direct combined)	0.438 [0.430, 0.446]
**GPC (combined)**	**0.702 [0.684, 0.720]**
GPC (direct combined)	0.698 [0.679, 0.716]
**Low-freq** (MAF $<$ 1%)	
Impute5 (EUR)	0.384 [0.354, 0.414]
RBM	0.061 [0.052, 0.070]
WGAN	0.009 [0.008, 0.009]
HMM	0.055 [0.050, 0.059]
HMM (direct)	0.087 [0.078, 0.096]
GPC	0.207 [0.191, 0.224]
GPC (direct)†	0.364 [0.337, 0.390]†
RBM (combined)	0.383 [0.352, 0.415]
WGAN (combined)	0.388 [0.358, 0.418]
HMM (combined)	0.386 [0.357, 0.416]
HMM (direct combined)	0.107 [0.096, 0.117]
**GPC (combined)**	**0.410 [0.380, 0.441]**
GPC (direct combined)	0.404 [0.373, 0.434]
**Rare** (MAF $<$ 0.1%)	
Impute5 (EUR)	0.115 [0.091, 0.139]
RBM	0.001 [0.000, 0.001]
WGAN	0.000 [0.000, 0.000]
HMM	0.001 [-0.001, 0.003]
HMM (direct)	0.004 [0.001, 0.007]
GPC	0.015 [0.008, 0.023]
GPC (direct)†	0.064 [0.051, 0.078]†
RBM (combined)	0.117 [0.094, 0.139]
WGAN (combined)	0.115 [0.093, 0.138]
HMM (combined)	0.112 [0.089, 0.136]
HMM (direct combined)	0.011 [0.007, 0.015]
**GPC (combined)**	**0.118 [0.095, 0.141]**
GPC (direct combined)	0.111 [0.087, 0.135]

S7 Table. **Summarized metrics for S6 Fig (UKBB).** Mean imputation performance across 10 bootstrapped replicates for all, low-frequency (MAF $<$ 1%), and rare (MAF $<$ 0.1%) SNPs. Best generative model using combined data is bolded; best using population-specific data only is marked with †.
