# Supplementary material for: GPC: An expressive and tractable deep generative model for genetic variation data
Source: PLoS Genet. 2026 Oct 6;22(10):e1012321. doi: 10.1371/journal.pgen.1012321 (PMC13652338; doi:10.1371/journal.pgen.1012321)
Supplement: Supplementary file 16 [file pgen.1012321.s016.pdf]

**Method**	**Mean r^2 [95% CI]**
**All SNPs**	
Impute5	0.749 [0.745, 0.753]
RBM	0.574 [0.570, 0.578]
WGAN	0.474 [0.472, 0.476]
HMM	0.325 [0.323, 0.326]
HMM (direct)	0.374 [0.373, 0.376]
GPC	0.515 [0.511, 0.518]
**GPC (direct)**	**0.594 [0.589, 0.598]**
**Low-freq** (MAF $<$ 1%)	
Impute5	0.626 [0.618, 0.633]
RBM	0.301 [0.290, 0.312]
WGAN	0.120 [0.116, 0.123]
HMM	0.081 [0.077, 0.085]
HMM (direct)	0.120 [0.118, 0.122]
GPC	0.215 [0.209, 0.222]
**GPC (direct)**	**0.344 [0.335, 0.352]**

S8 Table. **Summarized metrics for Fig 8 (high-coverage 1KG).** Mean imputation performance across 10 bootstrapped replicates for all and low-frequency (MAF $<$ 1%) SNPs. Best generative model is bolded.
