# Supplementary material for: GPC: An expressive and tractable deep generative model for genetic variation data
Source: PLoS Genet. 2026 Oct 6;22(10):e1012321. doi: 10.1371/journal.pgen.1012321 (PMC13652338; doi:10.1371/journal.pgen.1012321)
Supplement: Supplementary file 17 [file pgen.1012321.s017.pdf]

Non-European

Method	Mean r^2 [95% CI]
**All SNPs**	
Impute5 (EUR)	0.338 [0.335, 0.342]
RBM	0.565 [0.562, 0.568]
WGAN	0.462 [0.459, 0.466]
HMM	0.332 [0.330, 0.334]
HMM (direct)	0.383 [0.380, 0.385]
GPC	0.529 [0.527, 0.531]
GPC (direct)†	0.593 [0.590, 0.596]†
RBM (combined)	0.584 [0.580, 0.588]
WGAN (combined)	0.494 [0.489, 0.499]
HMM (combined)	0.412 [0.408, 0.415]
HMM (direct combined)	0.372 [0.370, 0.374]
GPC (combined)	0.548 [0.545, 0.552]
**GPC (direct combined)**	**0.584 [0.581, 0.586]**
**Low-freq** (MAF < 1%)	
Impute5 (EUR)	0.046 [0.042, 0.049]
RBM	0.296 [0.287, 0.304]
WGAN	0.114 [0.112, 0.116]
HMM	0.084 [0.080, 0.087]
HMM (direct)	0.129 [0.126, 0.132]
GPC	0.237 [0.234, 0.240]
GPC (direct)†	0.339 [0.335, 0.344]†
RBM (combined)	0.329 [0.321, 0.337]
WGAN (combined)	0.162 [0.155, 0.169]
HMM (combined)	0.126 [0.121, 0.132]
HMM (direct combined)	0.121 [0.118, 0.124]
GPC (combined)	0.266 [0.256, 0.276]
**GPC (direct combined)**	**0.331 [0.323, 0.338]**

African

Method	Mean r^2 [95% CI]
**All SNPs**	
Impute5 (EUR)	0.268 [0.264, 0.273]
RBM	0.552 [0.543, 0.562]
WGAN	0.367 [0.363, 0.371]
HMM	0.321 [0.319, 0.323]
HMM (direct)	0.369 [0.366, 0.371]
GPC	0.546 [0.539, 0.552]
GPC (direct)†	0.583 [0.574, 0.591]†
**RBM (combined)**	**0.563 [0.553, 0.574]**
WGAN (combined)	0.407 [0.398, 0.416]
HMM (combined)	0.368 [0.361, 0.375]
HMM (direct combined)	0.360 [0.356, 0.363]
GPC (combined)	0.556 [0.548, 0.564]
GPC (direct combined)	0.562 [0.552, 0.571]
**Low-freq** (MAF < 1%)	
Impute5 (EUR)	0.022 [0.017, 0.027]
RBM	0.339 [0.320, 0.359]
WGAN	0.082 [0.078, 0.086]
HMM	0.109 [0.105, 0.114]
HMM (direct)	0.147 [0.141, 0.153]
GPC	0.317 [0.299, 0.334]
GPC (direct)†	0.376 [0.355, 0.396]†
**RBM (combined)**	**0.350 [0.330, 0.371]**
WGAN (combined)	0.101 [0.093, 0.110]
HMM (combined)	0.131 [0.124, 0.137]
HMM (direct combined)	0.140 [0.135, 0.145]
GPC (combined)	0.325 [0.307, 0.344]
GPC (direct combined)	0.342 [0.320, 0.363]

S9 Table. **Summarized metrics for Fig 9 (high-coverage 1KG).** Mean imputation performance across 10 bootstrapped replicates for all and low-frequency (MAF < 1%) SNPs. Best generative model using combined data is bolded; best using population-specific data only is marked with †. In this experiment, GPC (direct)† performs better than models using combined data.
