# Supplementary material for: GPC: An expressive and tractable deep generative model for genetic variation data
Source: PLoS Genet. 2026 Oct 6;22(10):e1012321. doi: 10.1371/journal.pgen.1012321 (PMC13652338; doi:10.1371/journal.pgen.1012321)
Supplement: Supplementary file 18 [file pgen.1012321.s018.pdf]

Method	Admixed individuals All SNPs	Admixed individuals MAF < 1%	Full test set All SNPs	Full test set MAF < 1%
(a) 1KG				
Impute5 (real panel)	0.566 [0.561, 0.571]	0.140 [0.130, 0.150]	0.649 [0.643, 0.655]	0.298 [0.286, 0.310]
**GPC (direct)**	**0.537 [0.533, 0.541]**	**0.114 [0.106, 0.122]**	**0.593 [0.589, 0.598]**	**0.218 [0.208, 0.228]**
GPC (AG panel)	0.507 [0.503, 0.512]	0.075 [0.067, 0.083]	0.540 [0.536, 0.544]	0.132 [0.123, 0.140]
RBM	0.480 [0.476, 0.484]	0.046 [0.040, 0.052]	0.494 [0.491, 0.496]	0.066 [0.060, 0.072]
WGAN	0.440 [0.436, 0.444]	0.009 [0.008, 0.011]	0.445 [0.443, 0.447]	0.009 [0.009, 0.010]
HMM (direct)	0.428 [0.425, 0.432]	0.022 [0.019, 0.025]	0.441 [0.437, 0.444]	0.039 [0.033, 0.045]
HMM (AG panel)	0.401 [0.398, 0.404]	0.013 [0.011, 0.015]	0.409 [0.406, 0.413]	0.024 [0.018, 0.030]
(b) High-coverage 1KG, array-based				
Impute5 (real panel)	0.668 [0.651, 0.685]	0.420 [0.377, 0.464]	0.749 [0.745, 0.753]	0.626 [0.618, 0.633]
**GPC (direct)**	**0.558 [0.547, 0.569]**	**0.274 [0.241, 0.307]**	**0.594 [0.589, 0.598]**	**0.344 [0.335, 0.352]**
RBM	0.536 [0.521, 0.550]	0.239 [0.202, 0.276]	0.574 [0.570, 0.578]	0.301 [0.290, 0.312]
GPC (AG panel)	0.493 [0.484, 0.501]	0.176 [0.147, 0.205]	0.515 [0.511, 0.518]	0.215 [0.209, 0.222]
WGAN	0.456 [0.448, 0.465]	0.104 [0.089, 0.119]	0.474 [0.472, 0.476]	0.119 [0.116, 0.123]
HMM (direct)	0.367 [0.362, 0.372]	0.100 [0.085, 0.115]	0.374 [0.373, 0.376]	0.120 [0.118, 0.122]
HMM (AG panel)	0.325 [0.321, 0.329]	0.077 [0.064, 0.089]	0.325 [0.323, 0.326]	0.081 [0.077, 0.085]

S10 Table. **Imputation accuracy restricted to admixed individuals.** Mean r^2 [95% CI] over 10 bootstrap replicates, for the 212 haplotypes (106 individuals) of the held-out test split whose 1KG population is admixed: the four American populations (CLM, MXL, PEL, PUR) together with the two recently admixed African-ancestry populations (ASW, ACB). No model is retrained. These are the cosmopolitan models of the general 80/20 experiment, trained on the full training split rather than on population-specific data as in Section 2.6.2, and the models, reference panels and protocol are exactly those of that experiment; only the set of target haplotypes changes. The 1KG and high-coverage 1KG experiments use the same individuals and the same 80/20 split, so the subset is identical in both panels. Best generative model is bolded. Per-population counts are given in S11 Table.
