# Supplementary material for: GPC: An expressive and tractable deep generative model for genetic variation data
Source: PLoS Genet. 2026 Oct 6;22(10):e1012321. doi: 10.1371/journal.pgen.1012321 (PMC13652338; doi:10.1371/journal.pgen.1012321)
Supplement: Supplementary file 19 [file pgen.1012321.s019.pdf]

Dataset	Ancestry group	Constituent populations	Individuals
1KG	European (EUR)	CEU, FIN, GBR, IBS, TSI	502
	African (AFR)	ACB, ASW, ESN, GWD, LWK, MSL, YRI	661
	Admixed American (AMR)	CLM, MXL, PEL, PUR	347
	East Asian (EAS)	CDX, CHB, CHS, JPT, KHV	504
	South Asian (SAS)	BEB, GIH, ITU, PJL, STU	489
	Dual-labeled (EUR/AFR)	IBS, MSL	1
	Non-European (pooled)	AFR, AMR, EAS, SAS, dual-labeled	2,002
	**Total**		**2,504**
UKBB	European	British, Irish, any other white background	5,000
	African	African, Caribbean, any other Black background	5,000
	Non-European (pooled)	Indian, Pakistani, Bangladeshi, Chinese, any other Asian background, African, Caribbean, any other Black background	5,000
	**Total distinct**	(African and non-European share 1,538 individuals)	**13,462**

S11 Table. **Ancestry composition of the imputation datasets.** Counts are numbers of individuals. For 1KG, ancestry is the superpopulation code from the IGSR sample table, restricted to the 2,504 unrelated individuals, with the constituent population codes listed for each superpopulation. One individual carries two superpopulation labels and is shown as its own row; it is included in the non-European group, which is every individual not labeled European. The African target population is the AFR subset. For UKBB, ancestry is self-reported ethnic background (field 21000), and 5,000 individuals were sampled from each of the European, African and non-European groups. The non-European group includes individuals of African ancestry by design, so it overlaps the African group by 1,538 individuals and the three groups together contain 13,462 distinct individuals rather than 15,000.
