# Supplementary material for: GPC: An expressive and tractable deep generative model for genetic variation data
Source: PLoS Genet. 2026 Oct 6;22(10):e1012321. doi: 10.1371/journal.pgen.1012321 (PMC13652338; doi:10.1371/journal.pgen.1012321)
Supplement: Supplementary file 20 [file pgen.1012321.s020.pdf]

Response to Reviewers

Genetic Probabilistic Circuits

We thank both reviewers for their careful reading. Each comment is reproduced below with our response. Text added to the manuscript is shown *in red and indented*, and the corresponding edits appear in red in the revised manuscript.

Reviewer 1

1. Figure 1 is not sufficiently demonstrative for explaining the advantage of using HCLT. The second and third panels are too similar visually, so the reader may not clearly see why the tree-based structure is advantageous. Since the manuscript compares GPC/HCLT against HMM-based models in later sections, it would be helpful to include an explicit HMM chain structure in the figure and contrast it with the learned tree structure. This would make the difference between adjacent-SNP chain dependence and learned long-range tree dependence more intuitive, and would also help readers understand the topology shown in Figure 4.

We agree, and have redrawn Figure 1 with four panels: (a) six SNPs with a strong long-range correlation highlighted between X_2 and X_6; (b) the HMM chain over the latent variables; (c) the learned Chow–Liu tree; and (d) the HCLT, a tree over the latent variables. Panel (b) shows that under the chain there is no direct edge between Z_2 and Z_6, so the dependence must be routed through Z_3, Z_4 and Z_5. The same argument was added to Section 2.1, where the contrast between the two topologies is introduced, together with a forward reference to the visualization of a Chow–Liu tree learned from genetic data in Figure 5.

> *Figure 1 contrasts the two topologies on the same example. Under the chain (Figure 1b), Z_2 and Z_6 are four edges apart, so the dependence between X_2 and X_6 has no direct edge and must be carried through Z_3, Z_4 and Z_5. Each of these latent variables has a limited number of states, so some of the correlation is lost at every step. In the learned Chow–Liu tree (Figure 1c) the same pair is adjacent, and the dependence is represented by a single edge.*

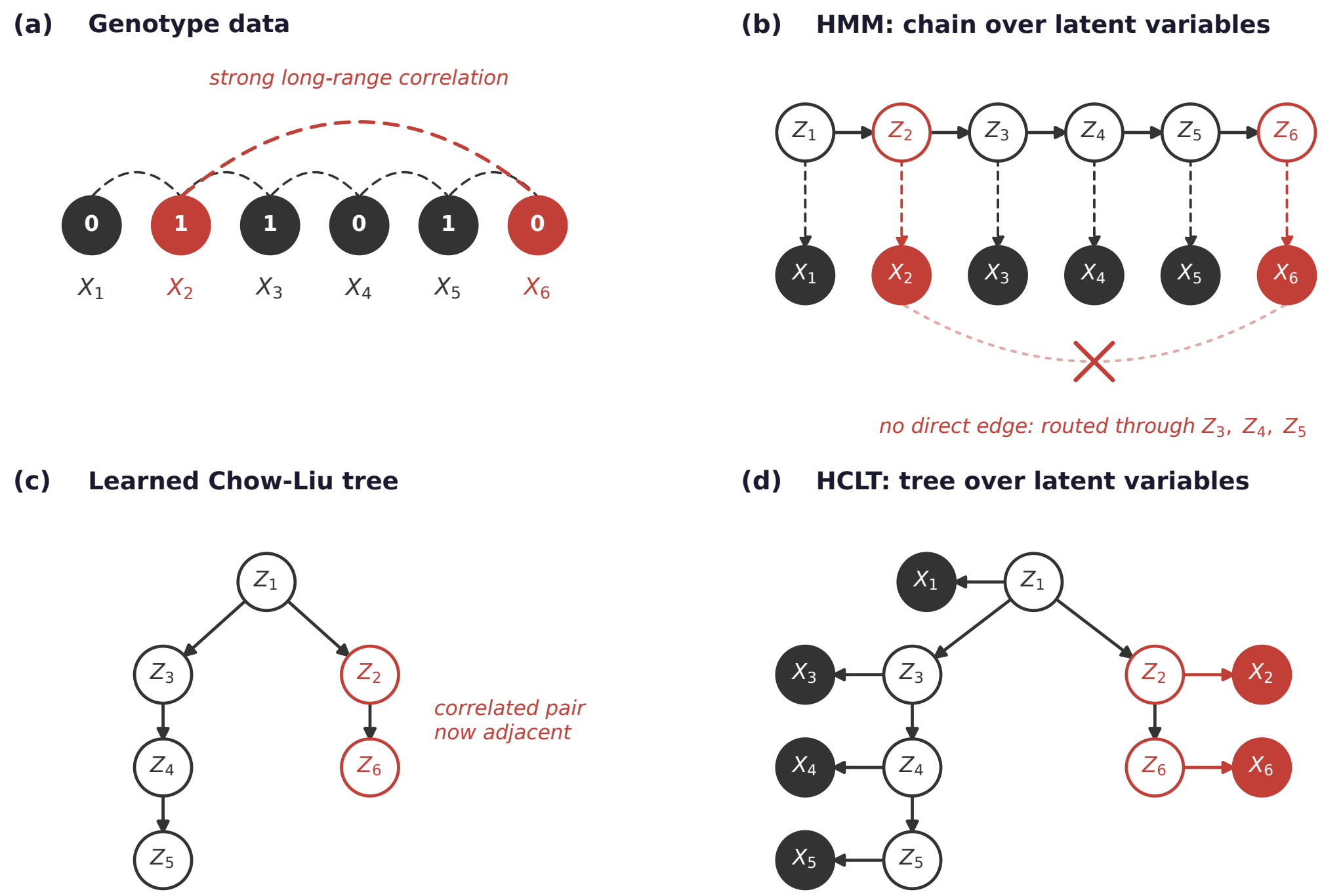

Revised Figure 1. The HMM and the HCLT on the same example.

2. It would be helpful to include a schematic workflow figure for the Section 2.6 imputation experiments. The current text describes two broad scenarios, but the workflow is not immediately clear. A figure could show how WGAN, RBM, and GPC are trained; how AGs are generated; how those artificial genomes are used as reference panels for Impute5; and how GPC direct imputation differs by computing conditional probabilities without generating artificial genomes as an intermediate step.

We thank the reviewer for the suggestion. We have added a schematic figure (Figure 2), referenced from the opening paragraph of Section 2.6. Panel (a) traces both routes from training haplotypes to the final r^2: the AG route (model, artificial genomes, reference panel, Impute5) and the direct route (model, exact conditional $P(X_{\text{miss}} \mid X_{\text{obs}})$, with no artificial genomes and no Impute5), together with the real-reference-panel benchmark that enters the same Impute5 step. Panel (b) shows that the three settings differ only in the training split, the test split, and which SNPs are masked.

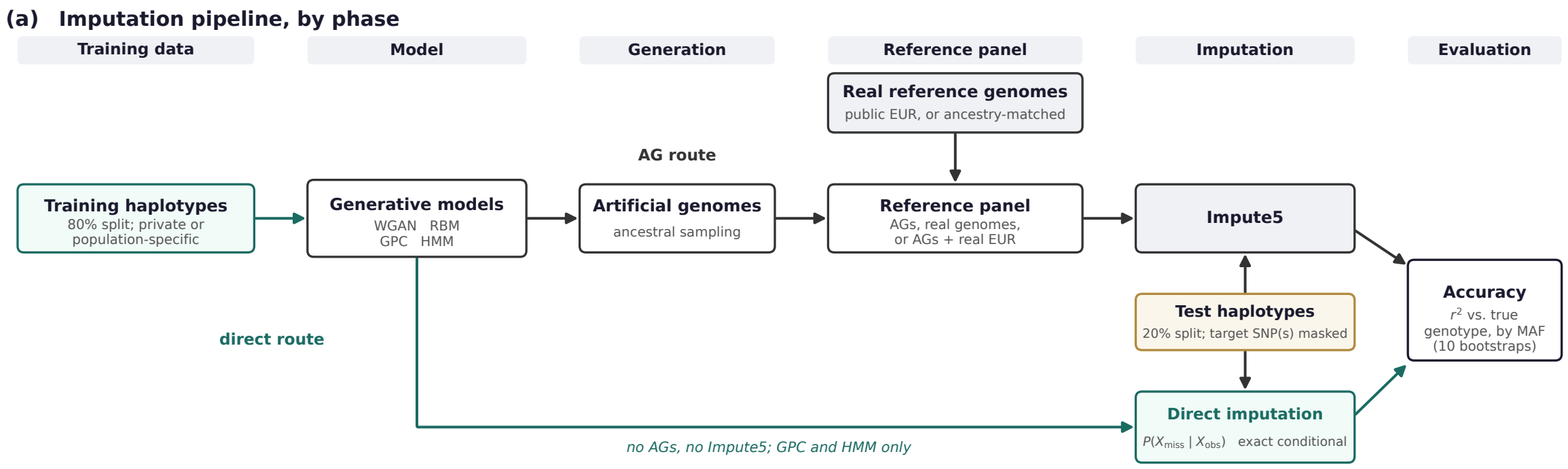

(b) The three settings differ only in the data splits and which SNPs are masked

Setting	Training data	Test data	SNPs imputed
General	80% of a cosmopolitan panel (1KG, UKBB)	20% held-out, same ancestries	one SNP at a time, all others observed
Population-specific	80% of the target population (African / non-European), optionally + real EUR	20% held-out of the target population	one SNP at a time, all others observed
Array-based	80% of high-coverage 1KG	20% held-out	12,551 non-array SNPs jointly, from 2,119 typed

New Figure 2. The two imputation routes and the three settings they are evaluated in.

3. The imputation r2 results for WGAN, RBM, and AG-based GPC are affected by Impute5 as an intermediate tool. Therefore, these results evaluate the combined pipeline of generative model plus artificial-genome reference panel plus Impute5, rather than the generative model alone. The manuscript should more clearly separate direct generative-model quality from downstream AG-plus-Impute5 utility. The current PCA, Wasserstein distance, LD, and privacy analyses are direct comparisons of generated artificial genomes, but the imputation analysis is partly dependent on Impute5.

We agree, and have added a paragraph at the start of Section 2.6 separating the two quantities and stating which results refer to which aspect of the evaluation. Figure 2 makes the same distinction visually.

> *The two routes measure different things, and we distinguish them throughout. The AG route evaluates a* pipeline *(generative model, then AG reference panel, then Impute5), so its accuracy reflects all three components, not the generative model alone. The direct route evaluates the generative model on its own, since the conditional $P(X_{\text{miss}} \mid X_{\text{obs}})$ is computed exactly from the model with no artificial genomes and no external imputation tool, and is available only to models that support tractable conditional inference. Direct evidence of model quality is therefore given by the held-out log-likelihoods of Table 1, the population-structure and LD comparisons of Figures 3 and 4, the privacy analysis of Table 2, and the direct imputation lines in the imputation figures; the AG panel lines instead quantify the downstream utility of the artificial genomes. The real-reference-panel Impute5 line bounds the contribution of the Impute5 component itself, since it applies the same tool to real reference genomes.*

4. The manuscript gives a clear and intuitive model-level explanation for why GPC/HCLT can improve over a chain-structured HMM: the learned tree topology can connect strongly correlated distant SNPs directly rather than propagating information through all intermediate SNPs. However, the model-level explanation for why GPC should outperform WGAN or RBM is less developed. The current discussion emphasizes tractability, likelihood evaluation, conditional inference,

and convergence monitoring, but it would be useful to explain more explicitly why WGAN/RBM may fail for genetic variation data, for example in terms of rare-variant modeling, mode collapse, memorization, poor conditional calibration, or difficulty preserving haplotype/LD structure.

We have expanded the Discussion to include such an explanation. The paragraph below was added to the Discussion, following the summary of our main findings.

> *Our results also suggest reasons why* WGAN *and* RBM *are less suited to this data. For* RBM, AA_{SYN} *near zero together with* AA_{TRUTH} *near one (Table 2) indicates that each synthetic haplotype lies closer to some individual real haplotype than to any other synthetic one, so the samples cluster around training points rather than covering the distribution. This is both a privacy risk and a failure to represent the full range of variation. For* WGAN*, uniformly high values on both components indicate the opposite behavior, artificial genomes that occupy a region largely disjoint from the real data. Both reproduce long-range LD reasonably but degrade at short range (Figure 4), whereas* GPC *is accurate at all scales. We believe two differences contribute to this gap. First, neither model supplies direct supervision on the dependence between particular pairs of SNPs: the* RBM *couples all SNPs through a single fully connected hidden layer that carries no notion of genomic position, and the* WGAN *critic returns a single scalar score per haplotype.* GPC *instead learns its tree from the pairwise mutual information of the data itself, so the dependence structure is fitted directly to the observed patterns of LD. Second, neither model admits a tractable likelihood, so convergence cannot be assessed objectively; the authors of the original works describe having to try multiple combinations of architectures and hyperparameter values, with some runs failing to converge, and assess convergence by visual overlap in principal component space.* GPC *is fit by EM and monitored by held-out log-likelihood, which supplies an objective stopping criterion.*

5. The scalability discussion is incomplete. The manuscript acknowledges that scaling GPC to full genome length remains challenging and that applying GPC independently to LD blocks may ignore cross-block LD. However, it does not clearly state the realistic working range of GPC in terms of SNP number, memory, and runtime beyond the demonstrated local regions. It also does not provide a systematic scalability comparison with WGAN, RBM, or genome-wide WGAN/CRBM variants. This is important because the demonstrated GPC applications appear to be around 10K to 15K SNP regions rather than whole-genome scale.

A new scaling study compares all three generative methods, and a new Section 4.5 in Materials and methods states the range explicitly. Training time and peak GPU memory were measured for GPC, RBM and WGAN across four haplotype counts and four SNP counts, drawn as random subsets of the high-coverage 1KG matrix, on an empty GPU, with every method run at the default parameters of its published training script. The results are now in Supplementary Figure S8.

GPC is the fastest to train at every point of the grid. On the full training set at $M = 12{,}287$ it takes 3.3 hours, against 22.8 for WGAN and 35.3 for RBM. The advantage is in the number of epochs rather than the cost of each: a GPC epoch is more expensive than an RBM epoch, but GPC converges in 5,000 epochs where RBM uses 100,000 and WGAN 2,001. This is the practical consequence of the convergence criterion discussed in Section 2.4: GPC exposes a held-out likelihood and can simply be trained until it flattens, whereas RBM and WGAN offer no such quantity, so their budgets are fixed in advance or chosen after the fact from visual inspection of

principal-component overlap. For peak memory, we find that RBM lowest at 1.3 GB, GPC at 4.7 GB and WGAN highest at 12.8 GB.

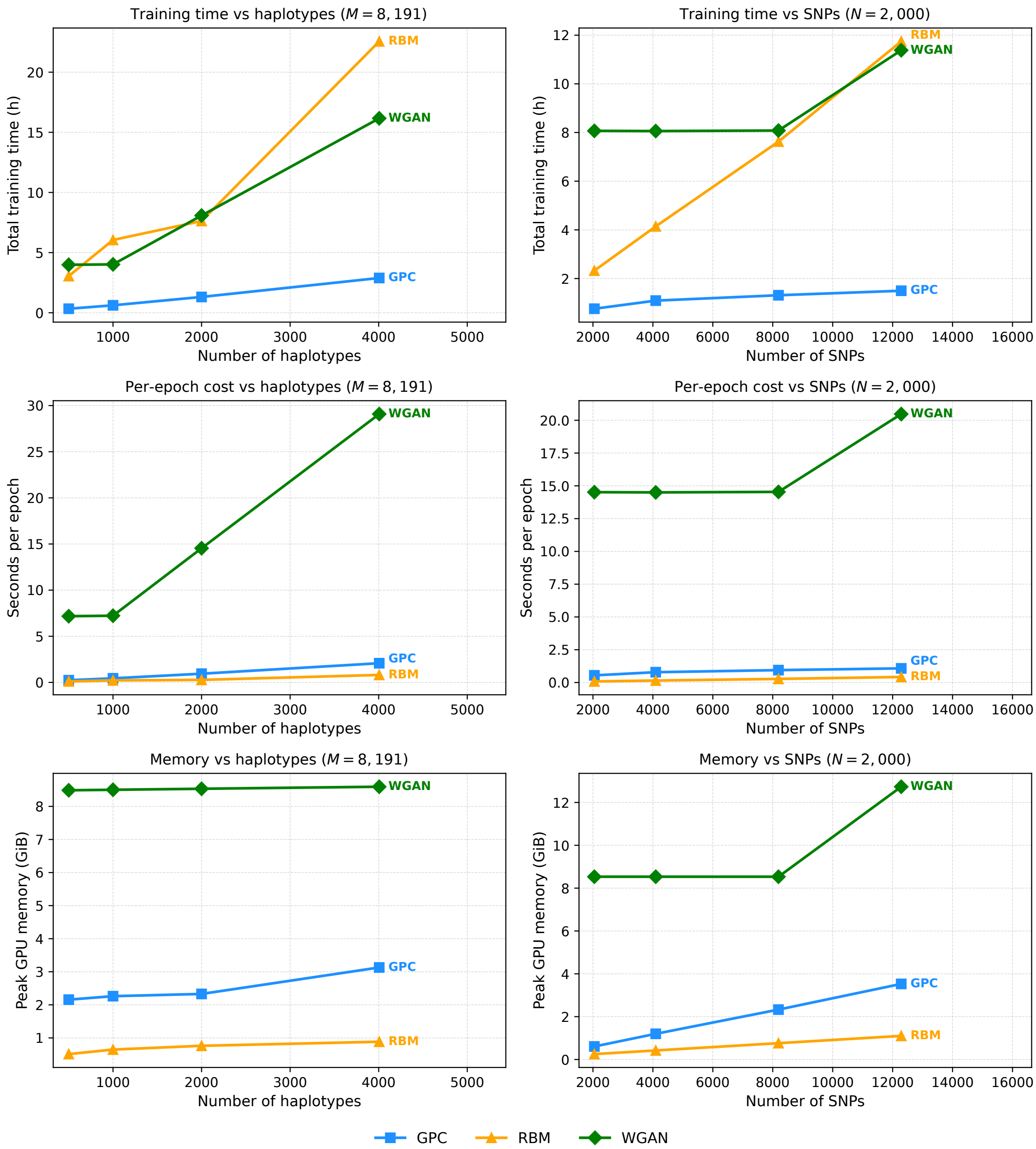

New Supplementary Figure S8. Training cost and peak memory against dataset size, for GPC, RBM and WGAN.

On the working range specifically, training is inexpensive: our largest model uses about 5 GB of GPU memory, and neither the latent-state count nor the epoch budget constrains region size. The limit is host memory while the Chow–Liu tree is built, which grows with the square of the number of SNPs, placing the practical working range at roughly 10,000 to 15,000 SNPs on a standard workstation. That is the scale of the regions used in the paper, and we now state it explicitly in Section 4.5. This ceiling follows from how the tree is currently constructed rather than from

the model: the tree-building step materializes an explicit graph over all pairs of SNPs, which is discarded as soon as the tree is found, and taking the tree directly from the pairwise statistics uses roughly seven times less memory, which corresponds to a region two to three times larger. We have checked on real data that the two routes return the same tree, but have not adopted the cheaper one for the results reported here.

On genome-wide WGAN and CRBM variants specifically, we address that under comment 6 below: those models target a different operating regime, at a much lower SNP density than the region studied here, so a runtime comparison against them would not be measuring the same task.

6. The manuscript mentions recent genome-wide WGAN and CRBM variants, but these are not included in the benchmark. If those methods are already designed for genome-wide synthetic genotype generation, the authors should clarify why they were not compared, or more explicitly state that the present comparison is limited to local genomic regions.

We have clarified the scope in the Discussion. Modeling a dense local region and modeling a whole genome are different problems: the genome-wide models operate at much lower SNP density and are typically built on genotyping-array frameworks, which are ascertained for common variation, whereas the regions studied here are sequence-based and substantially denser. The genome-wide WGAN and CRBM variants in particular are the same model families we already benchmark against, scaled up by tiling and convolution rather than redesigned. We also note that we cannot currently run GPC at genome-wide scale either.

The paragraph that we have added there also states that that none of the approaches cited (RBMs, VAEs, GANs, and diffusion models) can answer conditional queries: GANs do not define a tractable likelihood, RBMs have an intractable partition function, VAEs provide only a lower bound, and diffusion models require either approximations or a full multi-step denoising pass per condition.

7. How does GPC perform in admixed populations? Since the model is motivated partly by diverse and underrepresented populations, it would be useful to evaluate or at least discuss performance in explicitly admixed populations, where long-range ancestry-related correlations and local ancestry structure may be important.

We agree that admixed populations present an important setting. For these comparisons, we focus on the imputation experiments. Rather than training new models on a smaller admixed population which can make comparisons noisy, we re-evaluated the accuracy of our previously trained models on the subset of held-out individuals in 1KG from an admixed population. We examine the imputation models, trained on the cosmopolitan 80/20 split, for both the 1KG low and high coverage experiments. The models, reference panels, masking protocol and bootstrap procedure are all unchanged; only the set of held-out target haplotypes differs.

Our admixed cohort is the union of the four admixed American populations (CLM, MXL, PEL, PUR) and African-ancestry populations (ASW and ACB) resulting in 106 individuals in the held-out set (CLM 27, ACB 20, PEL 17, PUR 16, MXL 15, ASW 11 individuals). The 1KG and high-coverage 1KG experiments use the same individuals and the same split.

While accuracy across methods decreases in the admixed populations as expected, the ordering of the methods is unchanged with GPC (direct) remaining the most accurate generative model.

Mean r^2 on admixed individuals, with the full test set for reference.

**Method**	**Admixed** all	**Admixed** $< 1\%$	**Full test set** all	**Full test set** $< 1\%$
1KG				
Impute5 (real panel)	0.566	0.140	0.649	0.298
GPC (direct)	0.537	0.114	0.593	0.218
GPC (AG panel)	0.507	0.075	0.540	0.132
RBM	0.480	0.046	0.494	0.066
WGAN	0.440	0.009	0.445	0.009
HMM (direct)	0.428	0.022	0.441	0.039
HMM (AG panel)	0.401	0.013	0.409	0.024
High-coverage 1KG				
Impute5 (real panel)	0.668	0.420	0.749	0.626
GPC (direct)	0.558	0.274	0.594	0.344
RBM	0.536	0.239	0.574	0.301
GPC (AG panel)	0.493	0.176	0.515	0.215
WGAN	0.456	0.104	0.474	0.120
HMM (direct)	0.367	0.100	0.374	0.120
HMM (AG panel)	0.325	0.077	0.325	0.081

We have added a short note to each of the two corresponding imputation sections, 2.6.2 and 2.6.3, pointing to Supplementary Figure S7 and Supplementary Table S10 for the full results.

While our new results suggest GPC maintains its relative improvements in imputation accuracy for admixed populations, a more detailed understanding of the effectiveness of generative models in this setting is needed. These issues include investigating the impact of training on cosmopolitan panels relative to panels from an appropriately matched admixed population, assessing whether AGs generated in these populations are realistic in capturing patterns of population structure, LD, and local ancestry, and how these inferences are impacted by demographic history. Additionally, admixed populations present challenges for genome-wide AG generation due to the extent of long-range LD, so strategies such as applying models independently to LD blocks may be less effective in admixed populations. We have added this to the Discussion.

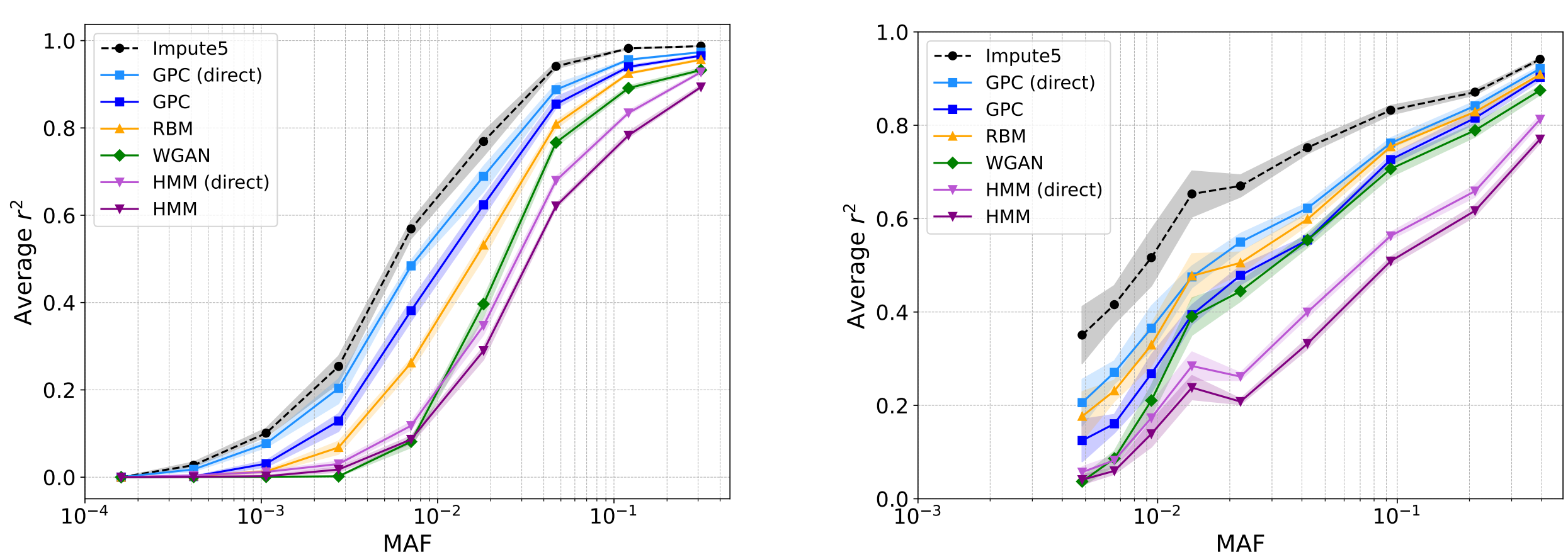

New Supplementary Figure S7. Imputation accuracy restricted to admixed individuals: 1KG (left) and high-coverage 1KG (right)

8. The population-specific imputation setup uses the category 'non-European'. This category

needs clarification. Does non-European include East Asian, South Asian, Native American/Latin American, African, and other groups together?

It does. We now state in Section 2.6.2 that "non-European (pooled)" denotes a heterogeneous group rather than a single population, comprising the African, admixed American, East Asian and South Asian superpopulations for 1KG and the corresponding self-reported groups for UKBB. Ancestry is taken from the IGSR sample table for 1KG and from self-reported ethnic background (field 21000) for UKBB. New Supplementary Table S11 gives the per-group counts.

New Supplementary Table S11. Ancestry composition of the imputation datasets, in individuals.

Dataset	Ancestry group	Constituent populations	Individuals
**1KG**	European (EUR)	CEU, FIN, GBR, IBS, TSI	502
	African (AFR)	ACB, ASW, ESN, GWD, LWK, MSL, YRI	661
	Admixed American (AMR)	CLM, MXL, PEL, PUR	347
	East Asian (EAS)	CDX, CHB, CHS, JPT, KHV	504
	South Asian (SAS)	BEB, GIH, ITU, PJL, STU	489
	Dual-labeled (EUR/AFR)	IBS, MSL	1
	Non-European (pooled)	AFR, AMR, EAS, SAS, dual-labeled	2,002
	**Total**		**2,504**
**UKBB**	European	British, Irish, any other white background	5,000
	African	African, Caribbean, any other Black background	5,000
	Non-European (pooled)	Indian, Pakistani, Bangladeshi, Chinese, any other Asian background, African, Caribbean, any other Black background	5,000
	**Total distinct**	(African and non-European share 1,538 individuals)	**13,462**

Reviewer 2

1. A major motivation for GPC is that classical models such as HMMs are limited by their chain-structured latent dependencies and therefore may fail to capture more complex dependency patterns. To more directly support this claim, it would be helpful to include an ablation in which the GPC framework is run with an HMM-like chain structure instead of the learned Chow–Liu tree structure. This comparison would isolate the contribution of the more flexible tree topology from other aspects of the GPC implementation, and would make it clearer how much of the performance gain is attributable specifically to the dependency structure.

We thank the reviewer for raising this point. The ablation already existed in the previous version, and we have made this clearer in three places. Section 2.3, where the baseline models are introduced, now states that the HMM is built in the same circuit framework as GPC and differs only in the latent graph, so every comparison between the two isolates the contribution of the topology. Section 4.4 gives the full specification: the same circuit library, the same number of latent states ($L = 128$), the same pseudocount (0.005), the same full-batch EM procedure and epoch budget, and identical training data. Section 2.7 notes the HMM's behavior on the privacy benchmark, where we also added it to Table 2.

On that benchmark the HMM attains $AA_{\text{TRUTH}} = 1.0000$ on both datasets, meaning no real haplotype ever has an artificial genome as its nearest neighbor, indicating that the HMM does not model the real data well enough (this is also evident from the PCA and LD decay plots).

2. The HMM baseline appears to be missing from the imputation benchmarks. Since one of the central claims is that GPC improves upon HMM-style modeling, including an HMM direct-imputation baseline would strengthen the comparison. Currently, HMM is mainly used in the likelihood and artificial-genome quality evaluations. However, lower held-out likelihood does not necessarily imply lower imputation accuracy, especially when the downstream task depends on conditional probabilities rather than the full joint distribution. Adding HMM direct imputation would therefore make the advantage of GPC over HMM more transparent.

We have added the chain-structured HMM to every imputation benchmark.

As expected, the relative ordering of GPC and HMM on the imputation task matches their ranking in terms of likelihoods, with the gap between the two models widening for rare variants. Comparing direct imputation using HMM and GPC isolates the impact of topology across settings. The HMM enters a large number of supplementary tables, so rather than reproduce them all we show below the three general-imputation settings with every method on the same axes, together with the corresponding rows lifted from the updated Supplementary Tables S4 (1KG), S6 (UKBB) and S8 (high-coverage 1KG).

Selected rows from the updated Supplementary Tables S4, S6 and S8, mean r^2 over all SNPs and at MAF $< 1\%$.

	**1KG**		**UKBB**		**High-cov. 1KG**	
**Method**	all	$< 1\%$	all	$< 1\%$	all	$< 1\%$
GPC (direct)	0.593	0.218	0.906	0.828	0.594	0.344
GPC (AG panel)	0.540	0.132	0.750	0.525	0.515	0.215
**HMM (direct)**	**0.441**	**0.039**	**0.468**	**0.138**	**0.374**	**0.120**
**HMM (AG panel)**	**0.409**	**0.024**	**0.414**	**0.099**	**0.325**	**0.081**

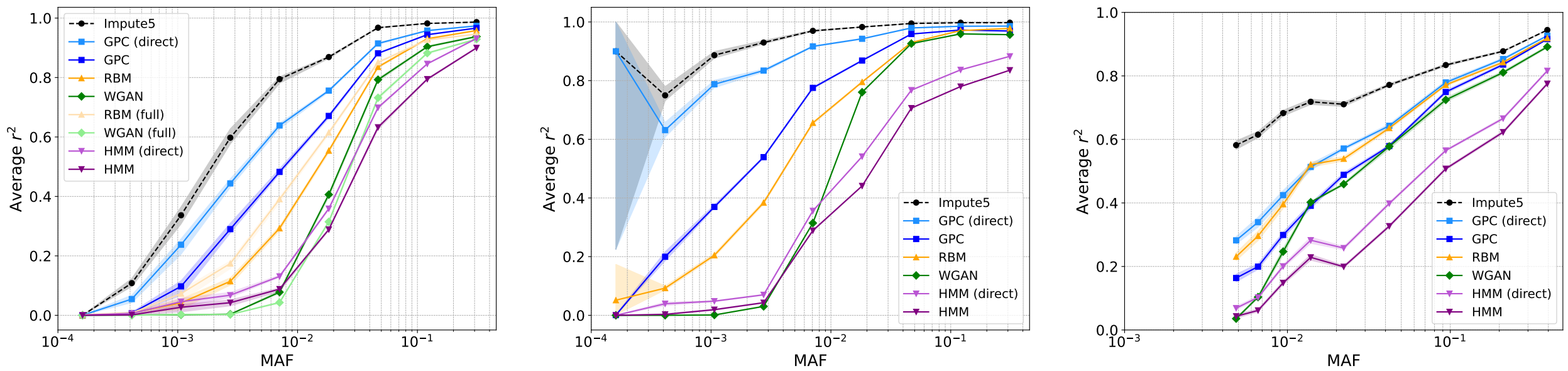

The same three settings as figures. 1KG (left), UKBB (center) and high-coverage 1KG (right), each now including the HMM for both imputation routes.

3. In Section 4.5.2 and the caption of Table S1, the PCA Wasserstein distance appears to be computed after refitting PCA separately for each method using the test set together with that method's artificial genomes. This gives a valid within-method diagnostic of whether the generated samples resemble the test samples in a method-specific PCA space. However, because each method is evaluated in a different PCA basis, with potentially different retained axes and scaling, the resulting Wasserstein distances are not directly comparable across methods. The authors should either clarify this limitation or recompute the PCA-based distances in a common coordinate system, for example by fitting PCA once on the real data or on the union of all methods and then projecting all generated samples into the same space.

We thank the reviewer for this observation and agree. We have recomputed all PCA distances in a common basis: a single PCA fit once on the union of all eight datasets (training set, test set and the six sets of artificial genomes), with every dataset projected into that same space. These are reported in the new Supplementary Table S2. We have kept the coupled-PCA values in Table S1, labeled as a within-method diagnostic, and rewritten the corresponding Methods paragraph to describe both bases (the section the reviewer cites has shifted from 4.5.2 to 4.6.2, because a new Section 4.5 was added in response to comment 4 below). The common basis is the one the PCA figures already use, so the reported distances correspond to the separation visible in those figures.

The chain-structured HMM is the setting most affected by the change of basis, though the rankings among the methods are otherwise largely preserved.

Supplementary Table S2 (new): PCA distances in a common basis.

Dataset		Truth	Indep	Markov	HMM	WGAN	RBM	GPC
**1KG**	PCA1-2	0.0026	0.2491	0.2260	0.0840	0.0029	**0.0026**	0.0059
	PCA3-4	0.0022	0.1402	0.1139	0.0129	0.0023	**0.0023**	0.0036
	PCA5-6	0.0022	0.1072	0.0855	0.0144	0.0024	**0.0023**	0.0024
**UKBB**	PCA1-2	0.0018	0.0856	0.0816	0.0390	0.0022	**0.0020**	0.0026
	PCA3-4	0.0020	0.0857	0.0720	0.0151	0.0023	**0.0020**	0.0024
	PCA5-6	0.0019	0.0658	0.0593	0.0273	0.0022	**0.0022**	0.0025

4. Scalability is presented as one of the practical advantages of GPC. The reported GPC training time, sampling time, and imputation time are informative, but it would be useful to provide comparable runtime and memory information for the baseline methods as well. This would help readers better understand the computational trade-offs between GPC, HMM, RBM, WGAN, and Impute5-based workflows. In addition, a scaling analysis showing how runtime and memory grow with the number of SNPs and the number of training samples would be valuable for users who may

want to apply GPC to larger genomic regions or whole chromosomes.

We agree, and have added a scaling study reporting runtime and memory for the baseline methods alongside GPC. Training time and peak GPU memory were measured for GPC, RBM and WGAN across four haplotype counts and four SNP counts, drawn as random subsets of the high-coverage 1KG matrix, on an empty GPU, with every method run at the default parameters of its published training script. The results are in Supplementary Figure S8.

The haplotype counts 500, 1,000, 2,000 and 4,006 double up to the full training set, and each is a nested random subsample so that the trend with N is not confounded by which haplotypes were drawn. The SNP counts 2,047, 4,095, 8,191 and 12,287 are set by WGAN, whose generator emits only lengths of the form latent size $\times 2^{12} - 1$ and cannot train on fewer than 8,191 SNPs; the two smaller sizes are therefore zero-padded up to 8,191, as its published pipeline does, which is why its cost is flat below that length.

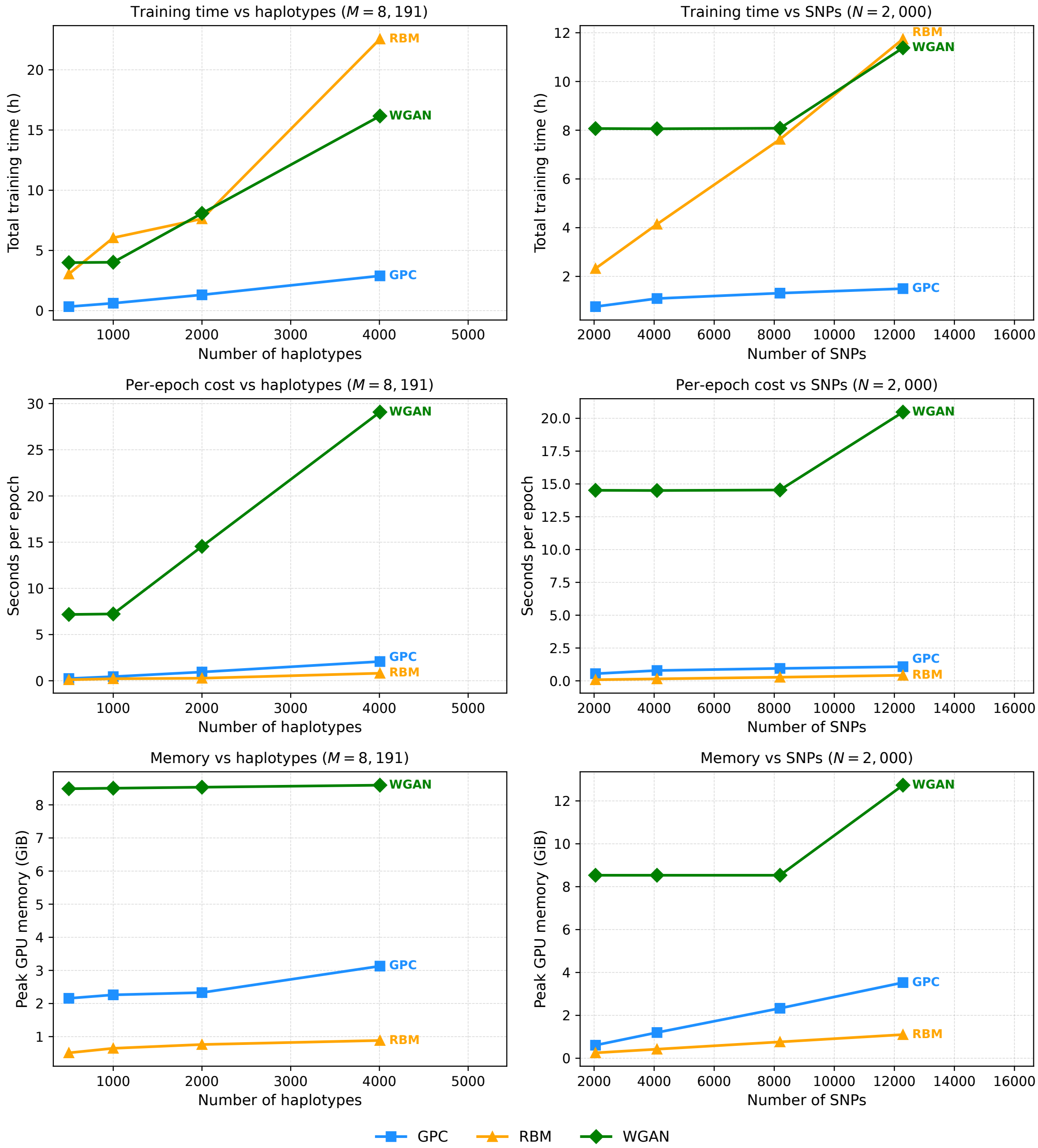

New Supplementary Figure S8. Training cost and peak memory against dataset size, for GPC, RBM and WGAN.

The trade-offs are as follows. GPC trains fastest at every point of the grid: on the full training set at $M = 12{,}287$ it takes 3.3 hours against 22.8 for WGAN and 35.3 for RBM. That advantage comes from the number of epochs rather than the cost of each one, since GPC converges in 5,000 epochs where RBM uses 100,000 and WGAN 2,001. Peak memory runs the other way, with RBM lowest at 1.3 GB, GPC at 4.7 GB and WGAN highest at 12.8 GB; WGAN's is nearly independent of dataset size because it is set by a fixed architecture. Cost grows close to linearly with the number of haplotypes for all three, while with the number of SNPs GPC is markedly flatter than RBM.

For users applying GPC to larger regions, the binding constraint is host memory while the Chow–

Liu tree is built, which grows with the square of the number of SNPs. This places the practical working range at roughly 10,000 to 15,000 SNPs on a standard workstation, which we now state explicitly in Section 4.5, and it is a property of how the tree is currently constructed rather than of the model. The step materializes an explicit graph over all pairs of SNPs that is discarded once the tree is found; taking the tree directly from the pairwise statistics instead uses roughly seven times less memory, and we have confirmed on real data that the two routes return the same tree. Extrapolating the same quadratic scaling, that would correspond to regions on the order of 40,000 SNPs, though we have not built a model at that size and have not adopted the cheaper route for the results reported here.
