# Supplementary material for: GPC: An expressive and tractable deep generative model for genetic variation data
Source: PLoS Genet. 2026 Oct 6;22(10):e1012321. doi: 10.1371/journal.pgen.1012321 (PMC13652338; doi:10.1371/journal.pgen.1012321)
Supplement: Peer review history

# Peer review: Decision Letter 0

**DOI:** 10.1371/journal.pgen.1012321.r001

**Authors:** Justin C. Fay, Yun Li

31 Jul 2026

PGENETICS-D-26-00468

GPC: An expressive and tractable deep generative model for genetic variation data

PLOS Genetics

Dear Dr. Anand,

Thank you for submitting your manuscript to PLOS Genetics. After careful consideration, we feel that it has merit but does not fully meet PLOS Genetics's publication criteria as it currently stands. Therefore, we invite you to submit a revised version of the manuscript that addresses the points raised during the review process.

Please submit your revised manuscript by Sep 29 2026 11:59PM. If you will need more time than this to complete your revisions, please reply to this message or contact the journal office at plosgenetics@plos.org. When you're ready to submit your revision, log on to https://www.editorialmanager.com/pgenetics/ and select the 'Submissions Needing Revision' folder to locate your manuscript file.

Please include the following items when submitting your revised manuscript:

* A letter that responds to each point raised by the editor and reviewer(s). You should upload this letter as a separate file labeled 'Response to Reviewers'. This file does not need to include responses to any formatting updates and technical items listed in the 'Journal Requirements' section below.

* A marked-up copy of your manuscript that highlights changes made to the original version. You should upload this as a separate file labeled 'Revised Manuscript with Track Changes'.

* An unmarked version of your revised paper without tracked changes. You should upload this as a separate file labeled 'Manuscript'.

If you would like to make changes to your financial disclosure, competing interests statement, or data availability statement, please make these updates within the submission form at the time of resubmission. Guidelines for resubmitting your figure files are available below the reviewer comments at the end of this letter.

As the corresponding author, your ORCID iD is verified in the submission system and will appear in the published article. PLOS supports the use of ORCID, and we encourage all coauthors to register for an ORCID iD and use it as well. Please encourage your coauthors to verify their ORCID iD within the submission system before final acceptance, as unverified ORCID iDs will not appear in the published article. Only the individual author can complete the verification step; PLOS staff cannot verify ORCID iDs on behalf of authors.

We look forward to receiving your revised manuscript.

Kind regards,

Yun Li

Academic Editor

PLOS Genetics

Justin Fay

Section Editor

PLOS Genetics

Aimée Dudley

Editor-in-Chief

PLOS Genetics

Anne Goriely

Editor-in-Chief

PLOS Genetics

**Additional Editor Comments (if provided):**

**Journal Requirements:**

If the reviewer comments include a recommendation to cite specific previously published works, please review and evaluate these publications to determine whether they are relevant and should be cited. There is no requirement to cite these works unless the editor has indicated otherwise.

1) We ask that a manuscript source file is provided at Revision. Please upload your manuscript file as a .doc, .docx, .rtf or .tex. If you are providing a .tex file, please upload it under the item type u2018LaTeX Source Fileu2019 and leave your .pdf version as the item type u2018Manuscriptu2019.

2) Please upload all main figures as separate Figure files in .tif or .eps format. For more information about how to convert and format your figure files please see our guidelines:

https://journals.plos.org/plosgenetics/s/figures

3) Please upload a copy of Figure Fig 1-8 which you refer to in your text on pages 4, 6, 7, 8, and 9. Or, if the figure is no longer to be included as part of the submission please remove all reference to it within the text.

4) We have noticed that you have uploaded Supporting Information files, but you have not included a list of legends. Please add a full list of legends for your Supporting Information files after the references list.

5) Please amend your detailed Financial Disclosure statement. This is published with the article. It must therefore be completed in full sentences and contain the exact wording you wish to be published.

i) State what role the funders took in the study. If the funders had no role in your study, please state: "The funders had no role in study design, data collection and analysis, decision to publish, or preparation of the manuscript."

ii) If any authors received a salary from any of your funders, please state which authors and which funders..

If you did not receive any funding for this study, please simply state: u201cThe authors received no specific funding for this work.u201d

**Reviewers' comments:**

Reviewer's Responses to Questions

**Comments to the Authors:**

**Please note here if the review is uploaded as an attachment.**

Reviewer #1: Comments and questions on the manuscript

This manuscript presents a solid and well-motivated methodological contribution for modeling genetic variation data through Genetic Probabilistic Circuits. I enjoyed reading the work and found the proposed framework both interesting and compelling. The manuscript is clearly written, easy to follow, and does a good job of explaining the motivation for moving beyond chain-structured HMM-based models toward a more flexible tree-structured representation. Overall, the method is technically sound, the empirical evaluations are extensive, and the proposed framework has clear potential utility for artificial genome generation, genotype imputation, and privacy-aware genomic data sharing.

The followings are my comments to the authors.

1. Figure 1 is not sufficiently demonstrative for explaining the advantage of using HCLT. The second and third panels are too similar visually, so the reader may not clearly see why the tree-based structure is advantageous. Since the manuscript compares GPC/HCLT against HMM-based models in later sections, it would be helpful to include an explicit HMM chain structure in the figure and contrast it with the learned tree structure. This would make the difference between adjacent-SNP chain dependence and learned long-range tree dependence more intuitive, and would also help readers understand the topology shown in Figure 4.

2. It would be helpful to include a schematic workflow figure for the Section 2.6 imputation experiments. The current text describes two broad scenarios, but the workflow is not immediately clear. A figure could show how WGAN, RBM, and GPC are trained; how AGs are generated; how those artificial genomes are used as reference panels for Impute5; and how GPC direct imputation differs by computing conditional probabilities without generating artificial genomes as an intermediate step.

3. The imputation r2 results for WGAN, RBM, and AG-based GPC are affected by Impute5 as an intermediate tool. Therefore, these results evaluate the combined pipeline of generative model plus artificial-genome reference panel plus Impute5, rather than the generative model alone. The manuscript should more clearly separate direct generative-model quality from downstream AG-plus-Impute5 utility. The current PCA, Wasserstein distance, LD, and privacy analyses are direct comparisons of generated artificial genomes, but the imputation analysis is partly dependent on Impute5.

4. The manuscript gives a clear and intuitive model-level explanation for why GPC/HCLT can improve over a chain-structured HMM: the learned tree topology can connect strongly correlated distant SNPs directly rather than propagating information through all intermediate SNPs. However, the model-level explanation for why GPC should outperform WGAN or RBM is less developed. The current discussion emphasizes tractability, likelihood evaluation, conditional inference, and convergence monitoring, but it would be useful to explain more explicitly why WGAN/RBM may fail for genetic variation data, for example in terms of rare-variant modeling, mode collapse, memorization, poor conditional calibration, or difficulty preserving haplotype/LD structure.

5. The scalability discussion is incomplete. The manuscript acknowledges that scaling GPC to full genome length remains challenging and that applying GPC independently to LD blocks may ignore cross-block LD. However, it does not clearly state the realistic working range of GPC in terms of SNP number, memory, and runtime beyond the demonstrated local regions. It also does not provide a systematic scalability comparison with WGAN, RBM, or genome-wide WGAN/CRBM variants. This is important because the demonstrated GPC applications appear to be around 10K to 15K SNP regions rather than whole-genome scale.

6. The manuscript mentions recent genome-wide WGAN and CRBM variants, but these are not included in the benchmark. If those methods are already designed for genome-wide synthetic genotype generation, the authors should clarify why they were not compared, or more explicitly state that the present comparison is limited to local genomic regions.

7. How does GPC perform in admixed populations? Since the model is motivated partly by diverse and underrepresented populations, it would be useful to evaluate or at least discuss performance in explicitly admixed populations, where long-range ancestry-related correlations and local ancestry structure may be important.

8. The population-specific imputation setup uses the category 'non-European'. This category needs clarification. Does non-European include East Asian, South Asian, Native American/Latin American, African, and other groups together?

Reviewer #2: The authors introduce Genetic Probabilistic Circuits (GPC), a deep generative model for genotype simulation and imputation. GPC generalizes hidden Markov models by allowing a more flexible tree-structured dependency among latent variables, while retaining a probabilistic interpretation and tractable inference. The authors show that GPC outperforms HMMs in likelihood-based comparisons and performs comparably to other deep generative models for artificial genome generation. They further demonstrate strong performance in genotype imputation, where GPC outperforms the benchmark methods in terms of SNP-level (r^2). Finally, the authors evaluate privacy using nearest-neighbor-based metrics and suggest that GPC achieves a favorable balance between utility and privacy. Overall, the manuscript is well written, technically interesting, and addresses an important problem in population genetics. I have the following comments.

1. A major motivation for GPC is that classical models such as HMMs are limited by their chain-structured latent dependencies and therefore may fail to capture more complex dependency patterns. To more directly support this claim, it would be helpful to include an ablation in which the GPC framework is run with an HMM-like chain structure instead of the learned Chow–Liu tree structure. This comparison would isolate the contribution of the more flexible tree topology from other aspects of the GPC implementation, and would make it clearer how much of the performance gain is attributable specifically to the dependency structure.

2. The HMM baseline appears to be missing from the imputation benchmarks. Since one of the central claims is that GPC improves upon HMM-style modeling, including an HMM direct-imputation baseline would strengthen the comparison. Currently, HMM is mainly used in the likelihood and artificial-genome quality evaluations. However, lower held-out likelihood does not necessarily imply lower imputation accuracy, especially when the downstream task depends on conditional probabilities rather than the full joint distribution. Adding HMM direct imputation would therefore make the advantage of GPC over HMM more transparent.

3. In Section 4.5.2 and the caption of Table S1, the PCA Wasserstein distance appears to be computed after refitting PCA separately for each method using the test set together with that method’s artificial genomes. This gives a valid within-method diagnostic of whether the generated samples resemble the test samples in a method-specific PCA space. However, because each method is evaluated in a different PCA basis, with potentially different retained axes and scaling, the resulting Wasserstein distances are not directly comparable across methods. The authors should either clarify this limitation or recompute the PCA-based distances in a common coordinate system, for example by fitting PCA once on the real data or on the union of all methods and then projecting all generated samples into the same space.

4. Scalability is presented as one of the practical advantages of GPC. The reported GPC training time, sampling time, and imputation time are informative, but it would be useful to provide comparable runtime and memory information for the baseline methods as well. This would help readers better understand the computational trade-offs between GPC, HMM, RBM, WGAN, and Impute5-based workflows. In addition, a scaling analysis showing how runtime and memory grow with the number of SNPs and the number of training samples would be valuable for users who may want to apply GPC to larger genomic regions or whole chromosomes.

**********

**Have all data underlying the figures and results presented in the manuscript been provided?**

Large-scale datasets should be made available via a public repository as described in the *PLOS Genetics*  data availability policy, and numerical data that underlies graphs or summary statistics should be provided in spreadsheet form as supporting information.

Reviewer #1: Yes

Reviewer #2: Yes

**********

PLOS authors have the option to publish the peer review history of their article (what does this mean?). If published, this will include your full peer review and any attached files.

If you choose “no”, your identity will remain anonymous but your review may still be made public.

**Do you want your identity to be public for this peer review?**  For information about this choice, including consent withdrawal, please see our Privacy Policy.

Reviewer #1: **Yes:**  zikun yang

Reviewer #2: No

[NOTE: If reviewer comments were submitted as an attachment file, they will be attached to this email and accessible via the submission site. Please log into your account, locate the manuscript record, and check for the action link "View Attachments". If this link does not appear, there are no attachment files.]

**Figure resubmission:**

**Reproducibility:**

To enhance the reproducibility of your results, we recommend that authors of applicable studies deposit laboratory protocols in protocols.io, where a protocol can be assigned its own identifier (DOI) such that it can be cited independently in the future. Additionally, PLOS ONE offers an option to publish peer-reviewed clinical study protocols. Read more information on sharing protocols at https://plos.org/protocols?utm_medium=editorial-email&utm_source=authorletters&utm_campaign=protocols

# Author comment: Author response to Decision Letter 1

**DOI:** 10.1371/journal.pgen.1012321.r002

20 Aug 2026

Attachment Submitted filename: gpc_response.pdf

# Peer review: Decision Letter 1

**DOI:** 10.1371/journal.pgen.1012321.r003

**Authors:** Justin C. Fay, Yun Li

17 Sep 2026

Dear Dr Anand,

We are pleased to inform you that your manuscript entitled "GPC: An expressive and tractable deep generative model for genetic variation data" has been editorially accepted for publication in PLOS Genetics. Congratulations!

Before your submission can be formally accepted and sent to production you will need to complete our formatting changes, which you will receive in a follow up email. Please be aware that it may take several days for you to receive this email; during this time no action is required by you. Please note: the accept date on your published article will reflect the date of this provisional acceptance, but your manuscript will not be scheduled for publication until the required changes have been made.

Once your paper is formally accepted, an uncorrected proof of your manuscript will be published online ahead of the final version, unless you’ve already opted out via the online submission form. If, for any reason, you do not want an earlier version of your manuscript published online or are unsure if you have already indicated as such, please let the journal staff know immediately at plosgenetics@plos.org.

In the meantime, please log into Editorial Manager at https://www.editorialmanager.com/pgenetics/, click the "Update My Information" link at the top of the page, and update your user information to ensure an efficient production and billing process. Note that PLOS requires an ORCID iD for all corresponding authors. Therefore, please ensure that you have an ORCID iD and that it is validated in Editorial Manager. To do this, go to ‘Update my Information’ (in the upper left-hand corner of the main menu), and click on the Fetch/Validate link next to the ORCID field.  This will take you to the ORCID site and allow you to create a new iD or authenticate a pre-existing iD in Editorial Manager.

If you have a press-related query, or would like to know about making your underlying data available (as you will be aware, this is required for publication), please see the end of this email. If your institution or institutions have a press office, please notify them about your upcoming article at this point, to enable them to help maximise its impact. Inform journal staff as soon as possible if you are preparing a press release for your article and need a publication date.

Thank you again for supporting open-access publishing; we are looking forward to publishing your work in PLOS Genetics!

Yours sincerely,

Yun Li

Academic Editor

PLOS Genetics

Justin Fay

Section Editor

PLOS Genetics

Aimée Dudley

Editor-in-Chief

PLOS Genetics

Anne Goriely

Editor-in-Chief

PLOS Genetics

www.plosgenetics.org

BlueSky: @plos.bsky.social

----------------------------------------------------

Comments from the reviewers (if applicable):

Reviewer's Responses to Questions

**Comments to the Authors:**

**Please note here if the review is uploaded as an attachment.**

Reviewer #1: I congratulate the authors on this thoughtful and substantial revision. They have carefully addressed my concerns and significantly strengthened the manuscript. I appreciate their considerable effort and am satisfied with the revised version.

Reviewer #2: The authors have addressed all my comments and I have no further concerns.

**********

**Have all data underlying the figures and results presented in the manuscript been provided?**

Large-scale datasets should be made available via a public repository as described in the *PLOS Genetics*  data availability policy, and numerical data that underlies graphs or summary statistics should be provided in spreadsheet form as supporting information.

Reviewer #1: Yes

Reviewer #2: Yes

**********

PLOS authors have the option to publish the peer review history of their article (what does this mean?). If published, this will include your full peer review and any attached files.

If you choose “no”, your identity will remain anonymous but your review may still be made public.

**Do you want your identity to be public for this peer review?**  For information about this choice, including consent withdrawal, please see our Privacy Policy.

Reviewer #1: **Yes:**  Zikun Yang

Reviewer #2: No

----------------------------------------------------

**Data Deposition**

If you have submitted a Research Article or Front Matter that has associated data that are not suitable for deposition in a subject-specific public repository (such as GenBank or ArrayExpress), one way to make that data available is to deposit it in the Dryad Digital Repository. As you may recall, we ask all authors to agree to make data available; this is one way to achieve that. A full list of recommended repositories can be found on our website.

The following link will take you to the Dryad record for your article, so you won't have to re‐enter its bibliographic information, and can upload your files directly:

http://datadryad.org/submit?journalID=pgenetics&manu=PGENETICS-D-26-00468R1

More information about depositing data in Dryad is available at http://www.datadryad.org/depositing. If you experience any difficulties in submitting your data, please contact help@datadryad.org for support.

Additionally, please be aware that our data availability policy requires that all numerical data underlying display items are included with the submission, and you will need to provide this before we can formally accept your manuscript, if not already present.

----------------------------------------------------

**Press Queries**

If you or your institution will be preparing press materials for this manuscript, or if you need to know your paper's publication date for media purposes, please inform the journal staff as soon as possible so that your submission can be scheduled accordingly. Your manuscript will remain under a strict press embargo until the publication date and time. This means an early version of your manuscript will not be published ahead of your final version. PLOS Genetics may also choose to issue a press release for your article. If there's anything the journal should know or you'd like more information, please get in touch via plosgenetics@plos.org.

# Editor report: Acceptance letter

**DOI:** 10.1371/journal.pgen.1012321.r004

**Authors:** Justin C. Fay, Yun Li

PGENETICS-D-26-00468R1

GPC: An expressive and tractable deep generative model for genetic variation data

Dear Dr Anand,

We are pleased to inform you that your manuscript entitled "GPC: An expressive and tractable deep generative model for genetic variation data" has been formally accepted for publication in PLOS Genetics! Your manuscript is now with our production department and you will be notified of the publication date in due course.

The corresponding author will soon be receiving a typeset proof for review, to ensure errors have not been introduced during production. Please review the PDF proof of your manuscript carefully, as this is the last chance to correct any errors. Please note that major changes, or those which affect the scientific understanding of the work, will likely cause delays to the publication date of your manuscript.

Soon after your final files are uploaded, unless you have opted out or your manuscript is a front-matter piece, the early version of your manuscript will be published online. The date of the early version will be your article's publication date. The final article will be published to the same URL, and all versions of the paper will be accessible to readers.

For Research Articles, you will receive an invoice from PLOS for your publication fee after your manuscript has reached the completed accept phase. If you receive an email requesting payment before acceptance or for any other service, this may be a phishing scheme. Learn how to identify phishing emails and protect your accounts at https://explore.plos.org/phishing.

Thank you again for supporting PLOS Genetics and open-access publishing. We are looking forward to publishing your work!

With kind regards,

Janani Seenivasan

PLOS Genetics

On behalf of:

The PLOS Genetics Team

Carlyle House, Carlyle Road, Cambridge CB4 3DN | United Kingdom

plosgenetics@plos.org | +44 (0) 1223-442823

plosgenetics.org | Twitter: @PLOSGenetics
